# Ventilation strategies and risk factors for intraoperative respiratory critical events and postoperative pulmonary complications in neonates and small infants: a secondary analysis of the NECTARINE cohort^☆^^☆^

**DOI:** 10.1016/j.bja.2024.12.038

**Published:** 2025-02-20

**Authors:** Alexander Fuchs, Nicola Disma, Thomas Engelhardt, Vanessa Marchesini, Thomas Riedel, Krisztina Boda, Walid Habre, Thomas Riva, NECTARINE Steering Committee, Francis Veyckemans, Katalin Virag, Tom G. Hansen, Karin Becke-Jakob, Pierre Harlet, Laszlo Vutskits, Suellen M. Walker, Jurgen C. de Graaff, Marzena Zielinska, Dusica Simic, the NECTARINE Group of the European Society of Anaesthesiology and Intensive Care Clinical Trial Network, Christian Breschan, Rudolf Likar, Manuela Platzer, Isole Edelman, Johanes Eger, Stefan Heschl, Brigitte Messerer, Maria Vittinghof, Ruth Kroess, Martina Stichlberger, David Kahn, Thierry Pirotte, Caroline Pregardien, Francis Veyckemans, France Stevens, Johan Berghmans, Annemie Bauters, Luc De Baerdemaeker, Stefan De Hert, Koen Lapage, Aliaksandra Parashchanka, Jurgen Van Limmen, Piet Wyffels, Julie Lauweryns, Nadia Najafi, Joris Vundelinckx, Diana Butković, Ivana Kerovec Sorić, Sandra Kralik, Ana Markić, Josip Azman, Josko Markic, Daniela Pupacic, Michal Frelich, Petr Reimer, René Urbanec, Petra Cajková, Vladimír Mixa, Yvona Sedláčková, Lenka Knoppová, Alena Zlámalová (neé Květoňová), Martin Vavřina, Jiří Žurek, Tom Hansen, Arash Afshari, Anders Bastholm Bille, Marguerite Ellekvist, Mari-Liis Ilmoja, Reet Moor, Reet Kikas, Merle Väli, Kariantti Kallio, Elisa Reponen, Pertti Suominen, Sami Suvanto, Raisa Vähätalo, Hannu Kokki, Merja Kokki, Jarkko Harju, Miia Kokkonen, Jenni Vieri, Tuula Manner, Catherine Amory, Hugues Ludot, Dina Bert, Juliette Godart, Anne Laffargue, Hervé Dupont, Benjamin Urbina, Catherine Baujard, Philippe Roulleau, Giuseppe Staiti, Maryline Bordes, Karine Nouette Gaulain, Yann Hamonic, François Semjen, Olivier Jacqmarcq, Caroline Lejus-Bourdeau, Cécile Magne, Léa Petry, Lilica Ros, Aurélien Zang, Mehdi Bennis, Bernard Coustets, Rose Fesseau, Isabelle Constant, Eliane Khalil, Nada Sabourdin, Noemie Audren, Thomas Descarpentries, Fanny Fabre, Aurélien Legrand, Emilie Druot, Gilles Orliaguet, Lucie Sabau, Lynn Uhrig, François de la Brière, Karin Jonckheer, Jean-Paul Mission, Lucia Scordo, Caroline Couchepin, Christophe Dadure, Pablo De la Arena, Laurent Hertz, Philippe Pirat, Chrystelle Sola, Myriam Bellon, Souhayl Dahmani, Florence Julien-Marsollier, Daphne Michelet, Veronique Depret-Donatien, Anne Lesage, Jost Kaufmann, Michael Laschat, Frank Wappler, Karin Becke, Lena Brunner, Karin Oppenrieder, Gregor Badelt, Karin Hochmuth, Bernhard Koller, Anita Reil, Sebastian Richter, Thomas Fischer, Anja Diers, Clemens Schorer, Andreas Weyland, Ruth Cohausz, Franz-Josef Kretz, Michaela Löffler, Markus Wilbs, Claudia Hoehne, Johanna Ulrici, Christiane Goeters, Armin Flinspach, Matthias Klages, Simone Lindau, Leila Messroghli, Kai Zacharowski, Christoph Eisner, Thomas Mueller, Daniel Richter, Melanie Schäfer, Markus Weigand, Sebastian Weiterer, Miriam Ochsenreiter, Michael Schöler, Tom Terboven, Isabel Eggemann, Sascha Haussmann, Nicolas Leister, Christoph Menzel, Uwe Trieschmann, Sirin Yücetepe, Susanna Keilig, Peter Kranke, Yvonne Jelting, Torsten Baehner, Richard Ellerkmann, Shahab Ghamari, Claudia Neumann, Martin Söhle, Pelagia Chloropoulou, Vagia Ntritsou, Pinelopi Papagiannopoulou, Eleana Garini, Afroditi Karafotia, Panagoula Mammi, Evangelia Bali, Despoina Iordanidou, Anna Malisiova, Artemis Polyzoi, Adelais Tsiotou, Erzsebet Sapi, Edgar Székely, Nandor Kosik, Veronika Maráczi, Janos Schnur, Judit Csillag, János Gál, Gergely Göbl, Balázs Hauser, András Petróczy, Gyula Tövisházi, Stuart Blain, Sarah Gallagher, Sinead Harte, Mandy Jackson, Emma Meehan, Zeenat Nawoor, Brendan O'Hare, Mark Ross, Daniela Lerro, Marinella Astuto, Chiara Grasso, Rita Scalisi, Giulia Frasacco, Elena Lenares, Roberto Leone, Maurizia Grazzini, Carmelo Minardi, Nicola Zadra, Gilda Cinnella, Antonella Cotoia, Dario Galante, Brita De Lorenzo, Beate Kuppers, Giulia Bottazzi, Fabio Caramelli, Maria Cristina Mondardini, Emanuele Rossetti, Sergio Picardo, Alessandro Vittori, Anna Camporesi, Edoardo Calderini, Laura Brigitta Colantonio, Simona Anna Finamore, Giuliana Anna Porro, Rachele Bonfiglio, Nicola Disma, Svetlana Kotzeva, Leila Mameli, Girolamo Mattioli, Camilla Micalizzi, Alessia Montaguti, Angela Pistorio, Clelia Zanaboni, Anna Guddo, Gerald Rogan Neba, Moreno Favarato, Bruno Guido Locatelli, Micol Maffioletti, Valter Sonzogni, Rossella Garra, Maria Sammartino, Fabio Sbaraglia, Andrea Cortegiani, Alessandra Moscarelli, Elena Attanasi, Simonetta Tesoro, Cristina Agapiti, Francesca Pinzoni, Cesare Vezzoli, Federico Bilotta, Arta Barzdina, Zane Straume, Anda Zundane, Laura Lukosiene, Irena Maraulaite, Ilona Razlevice, Bernd Schmitz, Stephanie Mifsud, Carolin Aehling, Celia Allison, Rients De Boer, Dina Emal, Markus Stevens, Marielle Buitenhuis, Jurgen de Graaff, Inge De Liefde, Andreas Machotta, Gail Scoones, Lonneke Staals, Jeremy Tomas, Anouk Van der Knijff-van Dortmont, Marianne Veldhuizen, David Alders, Wolfgang Buhre, Eva Schafrat, Jan Schreiber, Petronella Mari Vermeulen, Mark Hendriks, Sandra Lako, Marieke Voet-Lindner, Barbe Pieters, Gert-Jan Scheffer, Luc Tielens, Anthony R. Absalom, Margot Bergsma, Joke De Ruiter, Sascha Meier, Martin Volkers, Tjerk Zweers, Anne M. Beukers, Christa Boer, Jurgen Dertinger, Sandra Numan, Bas Van Zaane, Wenche B. Boerke, Nil Ekiz, Kristoffer Stensrud, Inger Marie Drage, Erik Ramon Isern, Alicja Bartkowska-Sniatkowska, Malgorzata Grzeskowiak, Magdalena Juzwa-Sobieraj, Jowita Rosada-Kurasińska, Artur Baranowski, Karina Jakubowska, Dorota Lewandowska, Magdalena Mierzewska-Schmidt, Piotr Sawicki, Magdalena Urban-Lechowicz, Pomianek Przemyslaw, Marzena Zielinska, Teresa Leal, Maria Soares, Pedro Pina, Sílvia Pinho, Maria Domingas Patuleia, Catarina Cruz Esteves, Helena Salgado, Maria João Santos, Rodica Badeti, Iulia Cindea, Loredana Oana, Adriana Gurita, Luminita Ilie, Gabriel Mocioiu, Radu Tabacaru, Irina Trante, Valentin Munteanu, Mihai Morariu, Emese Nyíri, Ivana Budic, Vesna Marjanovic, Biljana Drašković, Marina Pandurov, Jordanka Ilic, Ana Mandras, Zdenka Rados, Nikola Stankovic, Maja Suica, Sladjana Vasiljevic, Mirjana Knezevic, Irina Milojevic, Ivana Petrov, Selena Puric Racic, Dusica Simic, Irena Simic, Marija Stevic, Irena Vulicevic, Banská Bystrica, Barbora Cabanová, Miloslav Hanula, Jelena Berger, Darja Janjatovic, Špela Pirtovšek Štupnik, Dolores Méndez, Gema Pino, Paloma Rubio, Alberto Izquierdo, Silvia López, Cristina González Serrano, Jesús Cebrián, Ana Peleteiro, Pilar Del Rey de Diego, Ernesto Martínez García, Carolina Tormo de las Heras, Pablo Troncoso Montero, Celia Arbona, David Artés, Alicia Chamizo, Silvia Serrano, Montserrat Suarez Comas, Francisco Escribá, Cristina Auli, Osvaldo Pérez Pardo, Natalia Sierra Biddle, Ceferina Suárez Castaño, María Isabel Villalobos Rico, Susana Manrique Muñoz, Irene García Martínez, Nuria Montferrer Estruch, Elena Vilardell Ortíz, Rodrigo Poves-Álvarez, Ivan Kohn, Ulf Lindestam, Jarl Reinhard, Albert Castellheim, Kerstin Sandström, Sporre Bengt, Rainer Dörenberg, Peter Frykholm, Maria Garcia, Ann Kvarnström, Emma Pontén, Thomas Bruelisauer, Gabor Erdoes, Heiko Kaiser, Mathias Marchon, Thomas Riva, Stefan Seiler, Yann Bögli, Mirko Dolci, Carine Marcucci, Walid Habre, Isabelle Pichon, Laszlo Vutskits, Mattias Casutt, Martin Hölzle, Thomas Hurni, Martin Jöhr, Anna-Ursina Malär, Jacqueline Mauch, Thomas Erb, Karin Oeinck, Mine Akin, Gulsen Keskin, Yesim Senayli, Guner Kaya, Pinar Kendigelen, Ayse Çiğdem Tutuncu, Zehra Hatipoğlu, Dilek Özcengiz, Hale Aksu Erdost, Elvan Öçmen, Çimen Olguner, Hilmi Ayanoglu, Pelin Corman Dincer, Tumay Umuroglu, Mustafa Azizoglu, Handan Birbiçer, Nurcan Doruk, Aslı Sagun, Sibel Baris, Dmytro Dmytriiev, Sridevi Kuchi, Nuria Masip, Peter Brooks, Alison Hare, Nargis Ahmad, Michelle Casey, Sam De Silva, Nadine Dobby, Prakash Krishnan, L. Amaki Sogbodjor, Ellie Walker, Suellen Walker, Stephanie King, Katy Nicholson, Michelle Quinney, Paul Stevens, Andrew Blevin, Mariangela Giombini, Chulananda Goonasekera, Sadia Adil, Stephanie Bew, Carol Bodlani, Dan Gilpin, Stephanie Jinks, Nalini Malarkkan, Alice Miskovic, Rebecca Pad, Juliet Wolfe Barry, Joy Abbott, James Armstrong, Natalie Cooper, Lindsay Crate, John Emery, Kathryn James, Hannah King, Paul Martin, Stefano Scalia Catenacci, Rob Bomont, Paul Smith, Sara Mele, Alessandra Verzelloni, Philippa Dix, Graham Bell, Elena Gordeva, Lesley McKee, Esther Ngan, Jutta Scheffczik, Li-En Tan, Mark Worrall, Carmel Cassar, Kevin Goddard, Victoria Barlow, Vimmi Oshan, Khairi Shah, Sarah Bell, Lisa Daniels, Monica Gandhi, David Pachter, Chris Perry, Andrew Robertson, Carmen Scott, Lynne Waring, David Barnes, Sophie Childs, Joanne Norman, Robin Sunderland, Dowell Julia, Feijten Prisca, Harlet Pierre, Herbineaux Sarah, Leva Brigitte, Plichon Benoît, Virág Katalin

**Affiliations:** 9 Département d'Anaesthésie-Réanimation pédiatrique, Hôpital Jeanne de Flandre, CHRU de Lille, Lille, France; 10 Department of Medical Physics and Informatics, University of Szeged, Szeged, Hungary; 11 Department of Anaesthesia and Intensive Care - Paediatrics, Odense University Hospital, Department of Clinical Research - Anaesthesiology, University of Southern Denmark, Odense, Denmark; 12 Department of Anaesthesia and Intensive Care, Cnopf Children's Hospital/Hospital Hallerwiese, Nürnberg, Germany; 13 Research Department, European Society of Anaesthesiology, Brussels, Belgium; 14 Department of Anaesthesiology, Pharmacology, Intensive Care and Emergency Medicine, University Hospitals of Geneva, University of Geneva, Geneva, Switzerland; 15 Department of Paediatric Anaesthesia, Great Ormond St Hospital NHS Foundation Trust, London, UK; 16 Department of Anesthesia, Erasmus MC- Sophia Children's Hospital, Rotterdam, The Netherlands; 17 Department of Paediatric Anaesthesiology and Intensive Care, Wroclaw Medical University, Wroclaw, Poland; 18 Department of Pediatric Anesthesia and Intensive Care, University Children's Hospital, Medical Faculty University of Belgrade, Belgrade, Serbia; 20 Klinikum Klagenfurt, Austria; 21 Medical University Graz, Austria; 22 University Innsbruck, Austria; 23 Cliniques universitaires St Luc, Belgium; 24 Grand Hôpital de Charleroi, Belgium; 25 Queen Paola Children's Hospital, Belgium; 26 University Hospital Ghent, Belgium; 27 University Hospitals Leuven, Belgium; 28 Universitair Ziekenhuis Brussel (UZ Brussel), Belgium; 29 Ziekenhuis Oost-Limburg, Belgium; 30 Children's Hospital Zagreb, Croatia; 31 Rijeka University Hospital, Croatia; 32 University Hospital Split, Croatia; 33 Faculty Hospital Ostrava, Czech Republic; 34 Fakultni Nemocnice Motol, Czech Republic; 35 University Hospital Brno, Czech Republic; 36 Odense University Hospital, Denmark; 37 Rigshospitalet, Department of Anesthesia, Juliane Marie Center - University of Copenhagen, Denmark; 38 Tallinn Children's Hospital, Estonia; 39 Tartu University Hospital, Estonia; 40 Helsinki University Central Hospital, Finland; 41 Kuopio University Hospital, Finland; 42 Tampere University Hospital, Finland; 43 Turku University Hospital, Finland; 44 American Memorial Hospital CHU Reims, France; 45 CHRU de Lille Hôpital Jeanne de Flandre, France; 46 CHU Amiens Picardie, France; 47 CHU Bicetre, France; 48 CHU de Bordeaux, France; 49 CHU de Nantes, France; 50 CHU Nancy, France; 51 CHU Toulouse, France; 52 Hopital Armand Trousseau, France; 53 Hopital Couple Enfant CHU de Grenoble, France; 54 Hôpital Universitaire Necker Enfants Malades, France; 55 Hôpitaux Pédiatriques de Nice CHU Lenval, France; 56 Lapeyronie – CHU of Montpellier, France; 57 Robert Debré University Hospital, France; 60 Teaching Hospital of Caen, France; 61 Childrens Hospital Cologne, Germany; 62 Cnopf Childrens Hospital, Germany; 63 Klinik St. Hedwig, Germany; 64 Klinikum Kassel, Germany; 65 Klinikum Oldenburg, med. Campus der Universität, Germany; 66 Klinikum Stuttgart Olgahospital, Germany; 67 Universitaetsklinikum Leipzig, Germany; 68 University Hospital Münster, Germany; 70 University Hospital Frankfurt/Main, Germany; 71 University Hospital Heidelberg, Germany; 72 University Medical Centre Mannheim, Germany; 73 University Hospital of Cologne, Germany; 74 University of Wuerzburg, Germany; 75 University of Bonn, Germany; 76 Democritus University of Thrace, Greece; 77 "G. Gennimatas" General Hospital of Thessaloniki, Greece; 78 General Pediatric Hospital "Agia Sophia", Greece; 79 Hippokrateio General Hospital, Thessaloniki, Greece; 80 P & A Kyriakou Children Hospital, Greece; 81 Gottsegen Hungarian Institute of Cardiology, Hungary; 82 Heim Pal National Pediatric Institute, Hungary; 83 Semmelweis University, Hungary; 84 Children's Health Ireland – Crumlin, Ireland; 85 A.O.R.N Santobono Pausilipon, Italy; 86 A.O.U. Policlinico- Vittorio Emanuele Catania, Italy; 87 AOU A. Meyer, Italy; 88 Azienda Ospedaliera di Padova, Italy; 89 Azienda Ospedaliero Universitaria Ospedali Riuniti Di Foggia, Italy; 90 Azienda Ospedaliero-universitaria Pisana, Italy; 91 Azienda ospedaliero-universitaria Policlinico S.Orsola Malpighi, Italy; 92 Bambino Gesù Children's Hospital, IRCCS, Italy; 93 Children Hospital Vittore Buzzi, Italy; 94 Fondazione IRCCS Ca' Granda Ospedale Maggiore Policlinico, Italy; 95 Istituto Giannina Gaslini, Italy; 96 Ospedale dei bambini Di Cristina, Italy; 97 Ospedale G. Salesi, Italy; 98 Ospedale Papa Giovanni XXIII, Italy; 99 Fondazione Policlinico A.Gemelli IRCCS- Università Cattolica del Sacro Cuore- Roma, Italy; 100 Policlinico P. Giaccone, University of Palermo, Italy; 101 Section of Anesthesiology, Analgesia and Intensive Care, Italy; 102 Spedali Civili di Brescia, Italy; 103 University of Rome "Sapienza", Policlinico Umberto I, Rome, Italy; 104 Children Clinical University Hospital, Latvia; 105 Lithuanian University of Health Sciences Medical Academy, Lithuania; 106 Centre Hospitalier de Luxembourg, Luxembourg; 107 Mater Dei Hospital, Malta; 108 Amsterdam University Medical Center, University of Amsterdam, the Netherlands; 109 Erasmus MC- Sophia Children's Hospital, the Netherlands; 110 Haga Teaching Hospital, the Netherlands; 111 Leiden University Medical Center, the Netherlands; 112 MUMC, the Netherlands; 113 Radboudumc, the Netherlands; 114 Universitair Medisch Centrum Groningen, the Netherlands; 115 Amsterdam University Medical Center, VU University, the Netherlands; 116 University Medical Center Utrecht, the Netherlands; 117 Oslo University Hospital, Rikshospitalet, Norway; 118 Oslo University Hospital-Ullevål, Norway; 119 St. Olavs Hospital, Trondheim University Hospital, Norway; 120 Poznan University of Medical Sciences, Department of Pediatric Anesthesiology and Intensive Care, Poland; 121 Public Paediatric Teaching Hospital of Medical University of Warsaw, Poland; 122 Wroclaw Medical University, Poland; 123 Centro Hospitalar do Porto, Portugal; 124 Centro Hospitalar Lisboa Norte - Hospital de Santa Maria, Portugal; 125 Centro Hospitalar de Lisboa Central - Hospital D. Estefânia, Portugal; 126 Hospital de Braga, Portugal; 127 CHILDREN HOSPITAL 'LOUIS TURCANU", Romania; 128 Emergency Clinical Hospital of Constanta, Romania; 129 Emergency Hospital for Children Cluj Napoca, Romania; 130 Spitalul de copii "Maria Curie", Romania; 131 St. Maria" Children' s Hospital-Iasi", Romania; 132 Tirgu Mures Clinical Emergency Hospital, Romania; 133 Clinic for Anesthesiology and Intensive Therapy, Clinical Centre Nis, Medical Faculty University of Nis, Serbia; 134 Children and Youth Health Care Institute of Vojvodina, Serbia; 135 Mother and Child Healthcare Institute of Serbia, Serbia; 136 University Children s Hospital, Belgrade, Serbia; 137 University Children Hospital, Slovakia; 138 University Clinical Centre Ljubljana, Slovenia; 139 Hospital 12 de Octubre, Spain; 140 Hospital Universitari Parc Taulí Sabadell, Spain; 141 Hospital Universitario Donostia, Spain; 142 Hospital General Universitario Gregorio Marañón, Spain; 143 Hospital Infantil Universitario Niño Jesús, Spain; 144 Hospital Sant Joan De Déu, Spain; 145 Hospital Universitari i Politècnic la Fe, Spain; 146 Hospital Universitari Son Espases, Spain; 147 Hospital Universitario Marqués de Valdecilla, Spain; 148 Vall d'Hebron Barcelona Hospital Campus, Spain; 149 Hospital Clínico Universitario de Valladolid, Spain; 150 Astrid Lindgren Children's Hospital, Sweden; 151 Queen Silvia Children's Hospital, Sahlgrenska University Hospital, Sweden; 152 Uppsala University Hospital, Sweden; 153 Bern University Hospital, Switzerland; 154 Centre Hospitalier Universitaire Vaudois, Switzerland; 155 Geneva Children's Hospital, Switzerland; 156 Kantonsspital Luzern, Switzerland; 157 University Children's Hospital Basel, Switzerland; 158 Ankara Children's Health and Disease Haematology Oncology Training and Research Hospital, Turkey; 159 Cerrahpasa Medical School, Turkey; 160 Çukurova University, Turkey; 161 Dokuz Eylül University, Turkey; 162 Marmara University, Turkey; 163 Mersin University - Anaesthesiology, Turkey; 164 Ondokuz Mayis University, Turkey; 165 Vinnitsa National Medical University, Ukraine; 166 Alderhey Childrens Hospital, United Kingdom; 167 Chelsea & Westminster Hospital, United Kingdom; 168 Great Ormond St Hospital, United Kingdom; 169 Guy's and St Thomas's NHS Trust, United Kingdom; 170 Kings College Hospital, United Kingdom; 171 Leeds Children's Hospital, United Kingdom; 172 Nottingham University Hospital, United Kingdom; 173 Royal Aberdeen Children's Hospital, United Kingdom; 174 Royal Alexandra Childrens' Hospital, United Kingdom; 175 Royal Brompton Hospital, United Kingdom; 176 Royal Devon and Exeter NHS Foundation Trust, United Kingdom; 177 Royal Hospital for Sick Children Glasgow, United Kingdom; 178 Royal London Hospital, United Kingdom; 179 Royal Manchester Children's Hospital, United Kingdom; 180 Royal Victoria Infirmary, United Kingdom; 181 St Georges University Hospitals NHS Foundation Trust, United Kingdom; 182 European Society of Anaesthesiology, Brussels, United Kingdom; 1 Department of Anaesthesiology and Pain Medicine, Inselspital, Bern University Hospital, University of Bern, Bern, Switzerland; 2 Unit for Research in Anaesthesia, IRCCS Istituto Giannina Gaslini, Genoa, Italy; 3 Queen Elizabeth Hospital of Montreal Foundation, Department of Pediatric Anesthesia, Montreal Children's Hospital, Montreal, QC, Canada; 4 Department of Anaesthesia, Murdoch Children's Research Institute, Parkville, VIC, Australia; 5 Division of Paediatric Critical Care, Department of Paediatrics, Children's Hospital, Inselspital, Bern University Hospital, University of Bern, Bern, Switzerland; 6 Department of Medical Physics and Informatics, University of Szeged, Szeged, Hungary; 7 Faculty of Medicine, University of Geneva, Geneva, Switzerland

**Keywords:** intraoperative respiration, mechanical ventilation, neonate, neuromuscular blocking agent, paediatric, postoperative pulmonary complications, respiratory adverse events

## Abstract

**Background:**

Optimal ventilation strategies and use of neuromuscular blocking agents (NMBAs) in neonates and small infants undergoing anaesthesia remain unclear. We examined the association of perioperative ventilation strategies and administration of NMBAs on respiratory adverse events in the NEonate-Children sTudy of Anaesthesia pRactice IN Europe (NECTARINE) cohort.

**Methods:**

We performed a secondary analysis of NECTARINE, which included infants up to 60 weeks' postmenstrual age undergoing anaesthesia for surgical or diagnostic procedures. The primary endpoint was the association between ventilation mode and intraoperative respiratory adverse events. Secondary endpoints were use of NMBA, and 30-day postoperative pulmonary complications (PPCs).

**Results:**

The dataset comprised 5609 patients undergoing 6542 procedures. Pressure-controlled ventilation was the primary ventilation modality, accounting for 52.4% (*n*=3428) of cases. The incidence of intraoperative respiratory critical events was 20.7% (95% confidence interval [CI] 19.7–21.7%), while PPCs were observed in 17% of cases (95% CI 16.0–18.1%). Preanaesthesia respiratory conditions and NMBA use after tracheal intubation were associated with higher incidence of PPCs. Of the children receiving NMBAs, reversal was reported in 29.8%. The absence of reversal was associated with a higher incidence of PPCs, with a relative risk of 1.50 (95% CI 1.17–1.93). Conversely, NMBA reversal was associated with a reduced relative risk of 0.43 (95% CI 0.26–0.70).

**Conclusions:**

Regardless of ventilation strategy used, mechanical ventilation and baseline respiratory conditions were risk factors for a greater incidence of adverse respiratory events and PPCs. Reversal of NMBAs before tracheal extubation was significantly associated with reduced PPCs in neonates and should be routine clinical practice.

**Clinical trial registration:**

ClinicalTrials.gov (NCT02350348).

**Editor's key points**

• The impact of ventilation strategies and use of neuromuscular blocking agents (NMBAs) in neonates and small infants undergoing anaesthesia is unclear.
• This secondary analysis of the NECTARINE study assessed associations between perioperative ventilation strategies and administration of NMBAs on postoperative respiratory adverse events.
• Use of mechanical ventilation and baseline respiratory status were associated with a greater incidence of adverse respiratory events and postoperative pulmonary complications.
• Reversal of NMBAs before tracheal extubation, reported in only 30% of patients, was associated with reduced postoperative pulmonary complications.
• These findings identify opportunities for clinical practice guidelines for the systematic administration of reversal agents to reduce the risk of adverse events in neonates and infants.

Respiratory adverse events are the leading causes of perioperative morbidity and mortality in children,^1^^,^^2^ the risk of which is greater in neonates. In neonates, preanaesthesia respiratory support or airway and respiratory problems were also associated with a higher rate of intervention for intraoperative critical events.^3^ Given the significant efforts made in the last decade to promote lung-protective ventilation in the paediatric population, investigating the potential impact of intraoperative mechanical ventilation on patient outcomes would be of clinical relevance to optimise patient management.

Positive-pressure ventilation can be harmful to the lungs, even in healthy children.^4^^,^^5^ Therefore, selection of a ventilation modality that optimises gas exchange while safeguarding the lungs from injury during general anaesthesia is paramount in clinical practice. Modern anaesthesia ventilators now offer various ventilation modes, facilitated by advanced software systems that allow for sophisticated adjustments (e.g. pressure support). These capabilities facilitate patient-tailored ventilation strategies, optimising gas exchange and reducing respiratory morbidity.^6^ However, little is known about the ventilation modalities applied across Europe.

Another paradigm that has emerged recently and is reinforced in recent recommendations is the use of neuromuscular blocking agents (NMBAs) to facilitate tracheal intubation and ensure effective airway management.^7^^,^^8^ However, the incidence of residual neuromuscular block was found to be high in children, particularly when no reversal was administered.^9^ This raises questions about the role of reversing neuromuscular block in the occurrence of postoperative pulmonary complications (PPCs). This is even more concerning in neonates and infants, given their reduced functional residual capacity and respiratory reserve. Therefore, antagonism of nondepolarising NMBAs becomes paramount, particularly in this age group.^10^^,^^11^ Therefore, our aim was to delineate current clinical practices regarding ventilation strategies and use of NMBAs in neonates enrolled in the NECTARINE study. We also report associated respiratory critical events, morbidity, and mortality and attempted to identify potential associations with intraoperative ventilation modality or use of NMBA reversal.

## Methods

The ‘NEonate-Children sTudy of Anaesthesia pRactice IN Europe’ (NECTARINE) has been registered (ClinicalTrials.gov, identifier: NCT02350348) and the study protocol is available.^12^ NECTARINE was a multicentre prospective observational study in 165 centres in 31 European countries. After obtaining ethics approval for each centre, between March 2016 and January 2017, infants up to 60 weeks' postmenstrual age undergoing anaesthesia for surgical or diagnostic procedures were recruited. Further details have been published.^3^^,^^13^ This report follows the Strengthening the Reporting of Observational Studies in Epidemiology (STROBE) guidelines.^14^

### Variables

We screened the NECTARINE database and included all procedures with a documented perioperative ventilation strategy in the case report form (CRF). Preanaesthesia breathing conditions and concurrent respiratory and airway problems were recorded. Ventilation was recorded as either spontaneous, assisted, or controlled. For controlled ventilation, further classification was volume-controlled, pressure-controlled, pressure-regulated volume-controlled, or high-frequency oscillatory ventilation.

Respiratory critical events were defined in the CRF as either poor oxygenation (triggered by low SpO_2_ or PaO_2_ hypoxaemia) or hypocapnia or hypercapnia triggering an intervention. The intervention required to improve poor oxygenation or to correct end-tidal CO_2_, the time of occurrence (induction, maintenance, awakening or in PACU), number of events, SpO_2_ threshold triggering intervention (<90%, <85%, <80%), PaO_2_ value, and duration of hypoxaemia (in min) were recorded.

In the follow-up of 30 days after the last procedure, PPCs were captured in the CRF. PPCs included respiratory complications such as failure of weaning with prolonged ventilator support, need for re-intubation after being extubated, pleural effusion, pneumonia, pneumothorax or need for extracorporeal membrane oxygenation (ECMO). Mortality was recorded 30 days and 90 days after the last procedure.

### Outcomes

The primary outcome was the intraoperative ventilation strategy associated with intraoperative respiratory critical events. Secondary outcomes included use of NMBAs, NMBA reversal before tracheal extubation, 30-day PPCs, and 90-day mortality.

### Statistical methods

The primary study size determination for NECTARINE was based on the estimation of ∼5000 patients assuming an expected rate of severe perioperative critical events of ∼11% with a drop-out rate of 15%. The current secondary analysis was conducted on all available data from the NECTARINE database that included all procedures with a documented perioperative ventilation strategy in the CRF. Descriptive analyses were performed reporting categorical variables as absolute frequencies and valid percentages. Incidences are reported as percentages and 95% exact binomial confidence intervals (CIs). To control for several confounders, propensity score modelling was applied for intervention variables using adjustment with propensity scores. For binary variables, propensity score was calculated by a binary logistic regression with the given binary variable as dependent variable and many possible covariates measured before the intervention (sex, corrected age [gestational age + chronological age], weight, type of procedure, type of surgery, current respiratory and airway problems on day of anaesthesia, current respiratory condition at day of anaesthesia). For variables with multiple categories, propensity scores were calculated by multinomial logistic regression.^15^^,^^16^ After calculation of propensity scores, the potential overlap of their distributions was visually checked. Subsequently, we checked whether balance in the distribution of all covariates was achieved across intervention categories using statistical methods (analysis of covariance [ancova], logistic, and multinomial regression). Details of the propensity score analysis methods are provided in the Supplementary material.

Calculation of relative risk (RR) was done by a generalised linear model for repeated measures design using Poisson distribution for the dependent variable with log-link function, corrected for the propensity score. Results are presented as RR with 95% CI. Level of significance was set at 0.05. Statistical analysis was performed with R^17^ and SPSS version 29 (IBM, Armonk, NY, USA).

## Results

### Ventilation strategy

Of 6542 procedures screened, 6537 were included in the analysis. The main intraoperative ventilation strategy was pressure-controlled ventilation (52.4%, *n*=3428), followed by spontaneous ventilation without airway management (14.5%, *n*=950), volume-controlled ventilation (11.7%, *n*=764), pressure-regulated volume-controlled ventilation (7.0%, *n*=458), and spontaneous ventilation with airway management (5.8%, *n*=384).

### Risk factors for intraoperative respiratory critical events

Intraoperative respiratory critical events triggering an intervention were recorded in 1351 procedures (20.7%; 95% CI 19.7–21.7%). Most (61.4%, *n*=829) were attributable to hypoxaemia, and 38.6% (*n*=522) to hypocapnia or hypercapnia. Table 1 summarises risk factors for intraoperative respiratory critical events. Independent of the ventilation strategy, there was a strong association between all mechanical positive ventilation strategies through a tracheal tube and a higher incidence of hypoxaemia, hypocapnia, or hypercapnia. Administration of an NMBA after tracheal intubation was associated with alterations in hypocapnia or hypercapnia. Preanaesthesia respiratory conditions, including a condition on the day of anaesthesia requiring supplemental oxygen or invasive or noninvasive ventilation were also significantly associated with respiratory critical events. This association was also detected if the child had concomitant respiratory and airway problems.

**Table 1:** Univariable analysis of risk factors associated with intraoperative respiratory critical events adjusted for propensity score. Data are *n* (%) or relative risk (RR) with corresponding 95% confidence interval (95% CI). ASA, American Society of Anesthesiologists; CPAP, continuous positive airway pressure; HFOV, high-frequency oscillatory ventilation; NIV, noninvasive ventilation; NMBA, neuromuscular blocking agent.

	Hypoxaemia					Hypocapnia or hypercapnia				
	Yes		No		RR (95% CI)	Yes		No		RR (95% CI)
	Total	Value	Total	Value		Total	Value	Total	Value	
ASA physical status ≥3	2342	388 (16.6)	3877	355 (9.2)	1.19 (0.99–1.42)	2342	294 (12.6)	3877	188 (4.8)	1.62 (1.17–2.25)
Ventilation strategy (spontaneous without airway management as reference)										
Spontaneous with airway management	383	31 (8.1)	947	46 (4.9)	1.84 (1.18–2.88)	383	13 (3.4)	947	3 (0.3)	11.97 (3.09–46.45)
Assisted ventilation	516	67 (13.0)	947	46 (4.9)	2.72 (1.90–3.90)	516	20 (3.9)	947	3 (0.3)	12.86 (3.50–47.04)
Volume-controlled (VC)	762	99 (13.0)	947	46 (4.9)	2.49 (1.76–3.54)	762	69 (9.1)	947	3 (0.3)	26.24 (7.57–90.98)
Pressure-controlled (PC)	3414	504 (14.8)	947	46 (4.9)	2.61 (1.92–3.55)	3415	346 (10.1)	947	3 (0.3)	28.65 (8.41–97.62)
Pressure-regulated volume-controlled (PRVC)	457	69 (14.9)	947	46 (4.9)	2.78 (1.93–4.00)	457	67 (14.7)	947	3 (0.3)	41.35 (11.91–143.56)
Airway management (face mask as reference)										
Tracheal tube	4266	625 (14.7)	726	43 (5.9)	2.14 (1.50–2.92)	4266	432 (10.1)	726	4 (0.6)	14.91 (5.50–40.38)
Supraglottic airway	719	51 (7.1)	726	43 (5.9)	1.35 (0.92–2.00)	719	25 (3.5)	726	4 (0.6)	6.77 (2.33–19.60)
NMBA used (no NMBA as reference)										
NMBA before tracheal intubation	2831	376 (13.3)	2669	276 (10.3)	1.2 (0.997–1.44)	2831	287 (10.1)	2669	105 (3.9)	2.01 (1.66–2.67)
NMBA after tracheal intubation	801	166 (20.7)	2669	276 (10.3)	1.3 (0.944–1.79)	801	127 (15.9)	2669	105 (3.9)	2.76 (1.99–3.82)
Current respiratory and airway problems (no problems as reference)	1189	285 (24.0)	5234	535 (10.2)	1.99 (1.70–2.32)	1186	158 (13.3)	5022	354 (7.0)	1.76 (1.44–2.16)
Breathing condition at day of anaesthesia (no oxygen, no ventilation as reference)										
Supplemental oxygen	407	85 (20.9)	5027	497 (9.9)	1.665 (1.31–2.12)	407	56 (13.8)	5027	302 (6.0)	1.80 (1.33–2.42)
NIV with CPAP	173	48 (27.8)	5027	497 (9.9)	1.98 (1.44–2.71)	173	31 (17.9)	5027	302 (6.0)	2.23 (1.50–3.29)
Intubated, conventional ventilated	801	172 (21.5)	5027	497 (9.9)	1.49 (1.21–1.84)	802	122 (15.2)	5027	302 (6.0)	1.99 (1.55–2.55)
Intubated, ventilated with HFOV	58	21 (36.8)	5027	497 (9.9)	1.92 (1.15–3.19)	57	3 (5.3)	5027	302 (6.0)	0.73 (0.22–2.40)
ECMO (extracorporeal membrane oxygenation)	19	1 (5.3)	5027	497 (9.9)	0.38 (0.05–2.72)	19	0 (0.0)	5027	302 (6.0)	

### Postoperative pulmonary complications

The incidence of PPCs in the current cohort was 17% (95% CI 16.0–18.1%). Table 2 summarises the risk factors associated with respiratory complications encountered on follow-up 30 days after the procedure. While the incidence was lower during spontaneous and assisted ventilation than during any positive-pressure ventilation modality, any positive-pressure ventilation strategy with an airway device was associated with higher risk for occurrence of PPCs. Similar to intraoperative respiratory critical events, administration of an NMBA after tracheal intubation and a positive history of respiratory problems were associated with a higher risk for occurrence of PPCs. There was no evidence for a difference in PPCs based on positive end-expiratory pressure (RR 1.071 [95% CI 0.990–1.158]). Nevertheless, neonates and small infants who were left intubated after the anaesthesia procedure had a higher incidence of PPCs 27.4% *vs* 3.6% (RR 7.66 [95% CI 6.19–9.46]).

**Table 2:** Risk factors associated with postoperative pulmonary complications at 30 days follow-up after the procedure adjusted for propensity score. Data are *n* (%) or relative risk (RR) with corresponding 95% confidence interval (95% CI). ASA, American Society of Anesthesiologists; CPAP, continuous positive airway pressure; HFOV, high-frequency oscillatory ventilation; NIV, noninvasive ventilation.

	Yes		No		RR (95% CI)
	Total *n*	Value *n* (%)	Total *n*	Value *n* (%)	
ASA physical status ≥3	1603	309	3178	56	2.86 (1.96 4.19)
Ventilation strategy (spontaneous without airway management as reference)					
Spontaneous with airway management	312	12 (3.8)	740	16 (2.2)	2.5 (1.21–5.16)
Assisted	423	29 (6.9)	740	16 (2.2)	3.13 (1.73–5.66)
Volume-controlled	610	61 (10.0)	740	16 (2.2)	3.62 (2.12–6.18)
Pressure-controlled	2528	282 (11.2)	740	16 (2.2)	3.62 (2.21–5.92)
Pressure-regulated volume-controlled	347	45 (13.0)	740	16 (2.2)	4.71 (2.87–8.18)
Airway management (face mask as reference)					
Tracheal tube	3189	317 (9.9)	581	13 (2.2)	3.26 (1.88–5.67)
Supraglottic airway	594	20 (3.4)	581	13 (2.2)	2.48 (1.26–4.91)
Neuromuscular blocking agent (NMBA) used (not used as reference)					
NMBA provided before tracheal intubation	2215	176 (7.9)	2054	114 (5.6)	1.05 (0.84–1.31)
NMBA provided after tracheal intubation	530	166 (31.3)	2054	114 (5.6)	2.25 (1.70–2.98)
Current respiratory and airway problems at the day of anaesthesia (no problems as reference)					
Breathing condition at the day of anaesthesia (no oxygen, no ventilation as reference)					
Supplemental oxygen	286	62 (21.7)	4038	122 (3.0)	5.81 (4.32–7.83)
NIV with CPAP	117	45 (38.5)	4038	122 (3.0)	10.22 (7.52–13.89)
Intubated, conventional ventilated	519	206 (36.7)	4038	122 (3.0)	10.12 (7.84–13.07)
Intubated, ventilated with HFOV	31	18 (58.1)	4038	122 (3.0)	11.91 (7.81–18.17)
ECMO (extracorporeal membrane oxygenation)	5	4 (80.0)	4038	122 (3.0)	20.19 (12.26–33.25)

### Neuromuscular blocking agent reversal

An NMBA was used in 58.7% (*n*=3840/6537) of all procedures. Of note, in 78% of the cases, an NMBA was administered before tracheal intubation, while in 22% of the cases, an NMBA was administered afterwards. Table 3 summarises the different NMBAs administered in the cohort; rocuronium (33.5%) and atracurium (32.0%) were the most frequently used NMBAs.

**Table 3:** Number of patients receiving a neuromuscular blocking agent and nondepolarising NMBA reversal for extubation.

	*n*	%
Patients receiving a neuromuscular blocking agent (NMBA)	3840	100.0
Rocuronium	958	33.5
Atracurium	914	32.0
Cisatracurium	471	16.5
Succinylcholine	255	8.9
Mivacurium	147	5.1
Vecuronium	114	4.0

Patients receiving NMBA reversal	3585	100.0
No NMBA reversal	1894	71.1
Neostigmine	660	24.8
Sugammadex	111	4.2

For further analysis, we excluded patients already intubated at the time of anaesthesia, those who received airway management other than tracheal intubation, those who received suxamethonium for tracheal intubation, and those who remained intubated at the end of the procedure. Of these eligible 3183 children with a tracheal tube, 75.6% (*n*=2407/3183) received a nondepolarising NMBA intraoperatively, while 24.4% (*n*=776/3183) did not (Table 4). Only 29.8% of children receiving a nondepolarising NMBA received a reversal agent (*n*=716/2407), with neostigmine administered in most cases (85.6%), and sugammadex in only 14.4%.

**Table 4:** Respiratory complications at 30 days after the procedure for patients with tracheal intubation∗ adjusted for propensity score. Data are *n* (%) or relative risk (RR) and corresponding 95% confidence interval (95% CI). ∗Excluded patients who were left intubated after the procedure (*n*=99) and patients receiving succinylcholine as NMBA (*n*=255).

	Total	Yes	No	RR (95% CI)
All patients with tracheal intubation	3183	368 (11.6)	2815 (88.4)	—
No NMBA given (reference)	776	65 (8.4)	711 (91.6)	—
Nondepolarising NMBA without reversal	1691	285 (16.9)	1406 (83.1)	1.50 (1.17–1.93)
Nondepolarising NMBA with reversal	716	18 (2.5)	698 (97.5)	0.43 (0.26–0.70)

The incidence of PPCs at 30 days for patients extubated at the end of the procedure and receiving an NMBA is shown in Table 5. There was a higher incidence of PPCs in children who did not receive reversal. In contrast, a protective effect was observed for reversal of nondepolarising neuromuscular block with an RR of 0.43 (95% CI 0.26–0.70).

**Table 5:** Ventilation strategy and post-discharge follow-up at 30 and 90 days.

Variable	Total	Spontaneous ventilation (*n*=1334)		Assisted ventilation (*n*=518)	Controlled ventilation (*n*=4685)			
		No airway management	With airway management		Volume-controlled	Pressure-controlled	Pressure-regulated volume-controlled	High-frequency oscillatory ventilation
Data available for follow-up at Day 30	5215 (100.0)	760 (100.0)	330 (100.0)	448 (100.0)	629 (100.0)	2658 (100.0)	364 (100.0)	26 (100.0)
Discharged to home	4168 (79.9)	704 (92.6)	306 (92.7)	392 (87.5)	508 (80.8)	1976 (74.3)	279 (76.6)	3 (11.5)
Still in hospital	407 (7.8)	19 (2.5)	8 (2.4)	25 (5.6)	50 (7.9)	267 (10.0)	32 (8.8)	6 (23.1)
Discharged to another hospital	257(4.9)	19 (2.5)	9 (2.7)	15 (3.3)	15 (3.3)	169 (6.4)	21 (5.8)	3 (11.5)
Still in intensive care unit	278 (5.3)	13 (1.7)	7 (2.1)	12 (2.7)	12 (2.7)	176 (6.6)	19 (5.2)	10 (38.5)
Death	105 (2.0)	5 (0.7)	0 (0.0)	4 (0.9)	9 (1.4)	70 (2.6)	13 (3.6)	4 (15.4)
Data available for follow-up at day 90	4180 (100.0)	625 (100.0)	240 (100.0)	372 (100.0)	535 (100.0)	2035 (100.0)	301 (100.0)	18 (100.0)
Discharged to home between day 30 and 90	439 (10.5)	27 (4.1)	10 (3.9)	30 (8.1)	53 (9.9)	283 (9.9)	29 (9.6)	7 (38.9)
Still in hospital	219 (5.2)	10 (1.5)	4 (1.6)	9 (2.4)	30 (5.6)	137 (4.8)	21 (7.0)	8 (44.4)
Death	31 (0.7)	1 (0.2)	2 (0.8)	0 (0.0)	6 (1.1)	18 (0.9)	4 (1.3)	0 (0.0)
Data available for overall mortality	4285 (100.0)	668 (100.0)	256 (100.0)	376 (100.0)	544 (100.0)	2105 (100.0)	314 (100.0)	22 (100.0)
Yes	136 (3.2)	6 (0.9)	2 (0.8)	4 (1.1)	15 (2.8)	88 (4.2)	17 (5.4)	4 (18.2)

### Ancillary analyses

Table 5 shows the postoperative discharge follow-up at 30 and 90 days and the mortality rate associated with different ventilation strategies. At 30 days, 10% of children were still in the hospital with half of them in an ICU. A higher mortality rate was found in infants who were ventilated with high-frequency oscillatory ventilation, demonstrating greater severity of illness of these neonates.

While there was no evidence for an association between time of surgery and intraoperative respiratory events, there was a higher incidence of PPCs in children operated as emergency cases or at nighttime (data not shown). A modest but not clinically significant association was found between length of surgery or length of anaesthesia and presence of intraoperative respiratory adverse events, RR 1.002 (95% CI 1.002–1.003) and RR 1.001 (95% CI 1.001–1.001) for oxygen desaturation, and RR 1.004 (95% CI 1.003–1.005) and RR 1.002 (95% CI 1.001–1.002) for hypocapnia or hypercapnia, respectively.

A subanalysis was performed to investigate the potential difference between infants who had minimally invasive surgery and those who had open abdominal, thoracic or genito-urinary surgery (Supplementary material, Supplementary Table S8). Laparoscopy for abdominal surgery was associated with higher incidence of interventions for hypoxaemia and alterations in CO_2_. Minimally invasive thoracic surgery was associated with higher incidence of interventions for hypoxaemia. However, there was no evidence for a difference in the incidence of PPCs at 30 days between the two surgical techniques.

## Discussion

This secondary analysis explored ventilation strategies used in neonates and small infants in Europe to examine the potential association of perioperative ventilation strategies and administration of NMBAs on respiratory adverse events and PPCs. There was high variability in ventilation strategies used. Independent of the ventilation modality, mechanical ventilation was associated with a high rate of intraoperative respiratory critical events and PPCs, confirming respiratory system involvement in the high morbidity observed in this age group. Baseline respiratory condition was associated with a higher risk ratio for intraoperative respiratory critical events and PPCs. Administering an NMBA after tracheal intubation was associated with more interventions and a higher incidence of PPCs if not reversed at the end of the procedure. However, reversing neuromuscular block before tracheal extubation led to a reduced risk ratio for PPCs at 30 days.

### Ventilation modalities

The respiratory system of neonates and infants is characterised by stiff lungs with low compliance and increased airway resistance.^18^ In the present study, more than half of the children were ventilated with a pressure-controlled mode, characterised by a decelerating gas flow pattern that avoids increases in driving pressure with the subsequent increase in strain and potentially improves intrapulmonary gas distribution.^19^ Nevertheless, considering the changes in lung compliance that occur during anaesthesia and surgery, pressure-regulated volume control (PRVC) has been recommended as it combines the benefits of a decelerating flow with a targeted stable tidal volume, reducing the risk for lung damage and maintaining normocarbia.^19^ A meta-analysis including randomised controlled trials comparing both modes in neonates demonstrated a clinical benefit of PRVC over pressure-controlled ventilation with decreased incidence of bronchopulmonary dysplasia, pneumothorax, hypocarbia, and death.^20^

### Perioperative respiratory critical events and ventilation mode

We observed a high rate of intraoperative respiratory critical events with all positive-pressure ventilation modes, with the highest incidence observed during high-frequency oscillatory ventilation, reflecting a severe respiratory precondition before anaesthesia, which led to the choice of this ventilation mode. The RR for intraoperative hypoxaemia, hypocapnia, or hypercapnia was higher with all ventilation modalities than spontaneous ventilation without an airway device. Many factors might contribute to this increase as identified in the univariate analysis as potential predictors, such as the presence of an airway device (tracheal tube or supraglottic airway), history of respiratory problems after birth, concomitant respiratory or airway problems, use of NMBAs, type of surgical procedure, and whether the child was in hospital (admitted from the ward or ICU), reflecting the presence of comorbidities. These findings are in line with previous studies highlighting the higher incidence of severe intraoperative respiratory complications in neonates and infants^2^^,^^21^ as a consequence of their developmental respiratory physiology with low oxygen reserve and higher vulnerability for gas exchange impairment. The association between minimally invasive abdominal surgery and the incidence of interventions for hypoxaemia and CO₂ alterations further underscores the importance of adapting intraoperative mechanical ventilation to infants' lung physiology.

### Postoperative pulmonary complications at 30 days

The incidence of PPCs at 30 days associated with positive-pressure ventilation independent of the modality applied intraoperatively was high in the entire NECTARINE cohort. Conversely, the low rate observed with spontaneous ventilation without an airway device could be attributed to patient medical condition (i.e. fewer respiratory problems), or to the diagnostic or surgical procedure necessitating minimal or no respiratory support. Nevertheless, even in infants undergoing anaesthesia, positive-pressure ventilation was associated with a higher risk of ventilation-related complications along with other risk factors related to anaesthesia management, such as use of a tracheal tube and administration of NMBAs. Infants who remained intubated at the end of the procedure were more likely to experience PPCs. This suggests that if mechanical ventilation is necessary, the least invasive mode should be set according to the clinical status. A common procedure in the studied age cohort was inguinal hernial repair. A recent randomised controlled trial investigated sedation *vs* general anaesthesia for inguinal hernial repair with caudal block in small infants up to 3 months of age.^22^ Using caudal block with dexmedetomidine sedation, in 96.1% of patients in the sedation group, tracheal intubation could be avoided.

Another major finding of the present study is the strong association between an increase in PPCs at 30 days and respiratory system condition on the day of anaesthesia. This finding, along with identification of other risk factors such as emergency procedures and operating in the ICU, indicates that these children pose a challenge for anaesthesia management.

### Neuromuscular blocking agents and reversal

The association between NMBA use and increased incidence of intraoperative interventions and PPCs if not reversed should be viewed in the context of variable reports in adults. Use of NMBAs in patients with supraglottic airways can elevate the risk of PPCs. This finding suggests that NMBA use, rather than tracheal intubation, might be a significant factor influencing the incidence of PPCs in adults.^23^^,^^24^ There are mixed results in adults regarding the effectiveness of NMBA reversal in preventing PPCs.^25^, ^26^, ^27^ These inconsistencies could stem from limitations in study design or from pharmacological differences between neostigmine and sugammadex.^26^

In neonates and small infants, NMBAs are recommended to facilitate airway management and tracheal intubation.^7^^,^^8^ The results of this study revealed that only children who received an NMBA after tracheal intubation had a higher risk for intraoperative respiratory critical events and PPCs at 30 days. As a greater rate of intraoperative respiratory events and intraoperative interventions was observed in those who received an NMBA after intubation, the severity of their clinical condition and underlying respiratory conditions might have contributed to their increased susceptibility to complications. Nevertheless, we cannot exclude that the act of tracheal intubation itself, rather than the use of any drug, increases the risk of PPCs.^24^ This hypothesis is further corroborated by the higher risk for intraoperative respiratory adverse events and PPCs observed independently of the ventilation modality.

One of the most important findings of the present study is the beneficial effect of NMBA reversal on reducing the risk for PPCs at 30 days. While early reports emphasised the importance of reversing neuromuscular block in neonates and small infants because of their reduced respiratory reserve,^10^ there remains a lack of guidance on managing NMBA use in this population.^11^

Clear evidence exists for the advantages of NMBA use in adults,^28^ and perioperative NMBA management guidelines have been established.^27^ Despite the knowledge gap in this population, adopting practices already established in adults such as continuous monitoring of the depth of neuromuscular block and routine administration of NMBA reversal agents is imperative.^27^^,^^29^ The safety of sugammadex administration and its efficacy compared with neostigmine have been reported in infants and toddlers.^30^ Reversing neuromuscular block is important as it is well known that residual postoperative neuromuscular block contributes to increased risk of PPCs, including hypoxaemia, airway obstruction, and atelectasis, which can lead to re-intubation.^31^ Therefore, our results advocate for systematic monitoring of the depth of neuromuscular block and administration of NMBA reversal in neonates and small infants as a sound clinical practice for safe extubation.

### Mortality

The high mortality in this population is comparable to that reported in previous studies over the last decade.^32^ There was a difference in the incidence of mortality between the different ventilation modalities used during anaesthesia management. While a significant increase is expected in those ventilated with high-frequency oscillatory ventilation given that these children were extremely premature with low body weight and thus likely to have impaired lung function, the difference observed between spontaneous ventilation and positive-pressure ventilation could be attributed to factors including comorbidities, physical status, or complexity of the surgery.

### Limitations

This is a secondary analysis of observational data. Although we adjusted the data using propensity score analysis, there remains a potential risk of confounding, and we did not impute missing data. The identification of strong associations does not imply causality, but our results have the potential for identifying novel research questions with robust, high-quality RCTs to confirm these findings. This is particularly relevant for the association between reversal of neuromuscular block and prevention of PPCs.

Another limitation could be the relatively high number of missing values for 30-day morbidity and mortality. Although we did not impute for missing data, we performed a complete case analysis to decrease this bias, and thus the results obtained are based solely on the available data and do not account for the potential biases introduced by missing data. Nevertheless, the conclusions drawn remain of high clinical relevance, and we believe these missing values did not affect the validity of the results. Finally, it cannot be excluded that clinical practice might have changed over the last few years. Nevertheless, evidence for associations between ventilation strategy, NMBA reversal, and adverse respiratory outcomes is lacking in this high-risk population.

### Conclusions

These results suggest higher incidence of intraoperative respiratory critical events for positive-pressure ventilation compared with spontaneous ventilation with or without an airway device. The greater incidence of PPCs at 30 days and mortality observed with positive-pressure ventilation underscores the importance of tailoring ventilation strategy to respiratory function and needs of neonates. Among the identified risk factors for PPCs at 30 days, the absence of NMBA reversal in patients extubated directly after the procedure was associated with increased morbidity. Conversely, administration of NMBA reversal reduced such complications. There is a need to improve clinical practices related to monitoring depth of neuromuscular block in neonates and infants, and to establish clinical practice guidelines for the systematic administration of reversal agents to reduce the risk of adverse events in this vulnerable population.

## Authors’ contributions

Conception and design: AF, ND, WH, TR

Data analysis: KB

Data interpretation: all authors

Writing of the manuscript: AF, TR, WH

Review of the manuscript and final approval: all authors

## Declaration of interest

TE is a member of the associate editorial board of the *British Journal of Anaesthesia*. The other authors declare that they have no conflicts of interest.

## References

[1] von Ungern-Sternberg B.S., Boda K., Chambers N.A. (2010). Risk assessment for respiratory complications in paediatric anaesthesia: a prospective cohort study. Lancet.

[2] Habre W., Disma N., Virag K. (2017). Incidence of severe critical events in paediatric anaesthesia (APRICOT): a prospective multicentre observational study in 261 hospitals in Europe. Lancet Respir Med.

[3] Disma N., Veyckemans F., Virag K. (2021). Morbidity and mortality after anaesthesia in early life: results of the European prospective multicentre observational study, neonate and children audit of anaesthesia practice in Europe (NECTARINE). Br J Anaesth.

[4] Kneyber M.C., Zhang H., Slutsky A.S. (2014). Ventilator-induced lung injury. Similarity and differences between children and adults. Am J Respir Crit Care Med.

[5] Donn S.M., Sinha S.K. (2006). Minimising ventilator induced lung injury in preterm infants. Arch Dis Child Fetal Neonatal Ed.

[6] Keszler M. (2017). Mechanical ventilation strategies. Semin Fetal Neonatal Med.

[7] Disma N., Asai T., Cools E. (2024). Airway management in neonates and infants: European Society of Anaesthesiology and Intensive Care and British Journal of Anaesthesia joint guidelines. Br J Anaesth.

[8] Disma N., Asai T., Cools E. (2024). Airway management in neonates and infants: European Society of Anaesthesiology and Intensive Care and British Journal of Anaesthesia joint guidelines. Eur J Anaesthesiol.

[9] Ledowski T., O'Dea B., Meyerkort L., Hegarty M., von Ungern-Sternberg B.S. (2015). Postoperative residual neuromuscular paralysis at an Australian tertiary children's hospital. Anesthesiol Res Pract.

[10] Meakin G.H. (2008). Neuromuscular blocking drugs in infants and children. Contin Educ Anaesth Crit Care Pain.

[11] Honsel M., Giugni C., Brierley J. (2014). Limited professional guidance and literature are available to guide the safe use of neuromuscular block in infants. Acta Paediatr.

[12] European Society of Anaethesia and Intensive Care (ESAIC). Study information NECTARINE. Available from: https://esaic.org/study/nectarine/(accessed 18 July 2024).

[13] Fuchs A., Disma N., Virag K. (2022). Peri-operative red blood cell transfusion in neonates and infants: NEonate and Children audiT of Anaesthesia pRactice IN Europe: a prospective European multicentre observational study. Eur J Anaesthesiol.

[14] von Elm E., Altman D.G., Egger M., Pocock S.J., Gøtzsche P.C., Vandenbroucke J.P. (2007). The Strengthening the Reporting of Observational Studies in Epidemiology (STROBE) statement: guidelines for reporting observational studies. Lancet.

[15] Imbens G. (2000). The role of the propensity score in estimating dose-response functions. Biometrika.

[16] Spreeuwenberg M.D., Bartak A., Croon M.A. (2010). The multiple propensity score as control for bias in the comparison of more than two treatment arms. Med Care.

[17] R Core Team (2020). https://www.r-project.org.

[18] Trachsel D., Erb T.O., Hammer J., von Ungern-Sternberg B.S. (2022). Developmental respiratory physiology. Paediatr Anaesth.

[19] Habre W. (2010). Neonatal ventilation. Best Pract Res Clin Anaesthesiol.

[20] Klingenberg C., Wheeler K.I., McCallion N., Morley C.J., Davis P.G. (2017). Volume-targeted versus pressure-limited ventilation in neonates. Cochrane Database Syst Rev.

[21] Tay C.L., Tan G.M., Ng S.B. (2001). Critical incidents in paediatric anaesthesia: an audit of 10 000 anaesthetics in Singapore. Paediatr Anaesth.

[22] Bong C.L., Tan J., Lim S. (2019). Randomised controlled trial of dexmedetomidine sedation vs general anaesthesia for inguinal hernia surgery on perioperative outcomes in infants. Br J Anaesth.

[23] Hammer M., Santer P., Schaefer M.S. (2021). Supraglottic airway device versus tracheal intubation and the risk of emergent postoperative intubation after general anaesthesia in adults: a retrospective cohort study. Br J Anaesth.

[24] Hunter J.M., Aziz M.F. (2021). Supraglottic airway versus tracheal intubation and the risk of postoperative pulmonary complications. Br J Anaesth.

[25] Grosse-Sundrup M., Henneman J.P., Sandberg W.S. (2012). Intermediate acting non-depolarizing neuromuscular blocking agents and risk of postoperative respiratory complications: prospective propensity score matched cohort study. BMJ.

[26] Liu H.M., Yu H., Zuo Y.D., Liang P. (2023). Postoperative pulmonary complications after sugammadex reversal of neuromuscular blockade: a systematic review and meta-analysis with trial sequential analysis. BMC Anesthesiol.

[27] Fuchs-Buder T., Romero C.S., Lewald H. (2023). Peri-operative management of neuromuscular blockade: a guideline from the European Society of Anaesthesiology and Intensive Care. Eur J Anaesthesiol.

[28] Lundstrom L.H., Duez C.H.V., Norskov A.K. (2018). Effects of avoidance or use of neuromuscular blocking agents on outcomes in tracheal intubation: a Cochrane systematic review. Br J Anaesth.

[29] Thilen S.R., Weigel W.A., Todd M.M. (2023). 2023 American Society of Anesthesiologists practice guidelines for monitoring and antagonism of neuromuscular blockade: a report by the American Society of Anesthesiologists task force on neuromuscular blockade. Anesthesiology.

[30] Franz A.M., Chiem J., Martin L.D., Rampersad S., Phillips J., Grigg E.B. (2019). Case series of 331 cases of sugammadex compared to neostigmine in patients under 2 years of age. Paediatr Anaesth.

[31] Murphy G.S., Brull S.J. (2010). Residual neuromuscular block: lessons unlearned. Part I: definitions, incidence, and adverse physiologic effects of residual neuromuscular block. Anesth Analg.

[32] de Graaff J.C., Johansen M.F., Hensgens M., Engelhardt T. (2021). Best practice & research clinical anesthesiology: safety and quality in perioperative anesthesia care. Update on safety in pediatric anesthesia. Best Pract Res Clin Anaesthesiol.

