# Supplementary material for: Ventilation strategies and risk factors for intraoperative respiratory critical events and postoperative pulmonary complications in neonates and small infants: a secondary analysis of the NECTARINE cohort☆
Source: Br J Anaesth. 2025 Feb 20;135(5):1528–36. doi: 10.1016/j.bja.2024.12.038 (PMC12597469; doi:10.1016/j.bja.2024.12.038)
Supplement: Multimedia component 1 [file mmc1.docx]

**Perioperative ventilation and NMBA in neonates - Supplemental materials**

**S1. Extended description of propensity score estimation.**

To compare non-randomized treatment strategies, propensity score (PS) methods may reduce the effects of confounding in observational studies. Rosenbaum and Rubin defined the PS^1^ as the conditional probability of assignment to a particular treatment given observed baseline covariates.

*Selecting covariates for PS.* All possible baseline covariates were measured before the intervention (sex, corrected age (gestational age + chronological age), weight, type of procedure, type of surgery, current respiratory and airway problems on the day of anaesthesia, breathing condition on the day of anaesthesia). During PS modelling, some of the covariates were omitted. A list of covariates used is in supplementary table 1.

*Estimation of the PS.* When there were two interventions (binary variables), the PS was estimated by binary logistic regression with the intervention as dependent variables and the covariates as independent variables. When there were more than two interventions (nominal variables with >2 categories), the method described by Imbens^2^ was used. Multiple PSs were calculated by a multinomial logistic regression with the intervention (e.g. ventilation strategy) as the dependent variable and the covariates as independent variables. Multinomial logistic regression calculates as many propensity scores as many categories of the dependent variables. As these scores add up to 1, only N-1 out of N multiple PSs are needed for further analysis.

*Checking for overlap of the distributions*. After the PS(s) were calculated, the overlap of their distribution was visually checked. Since it is important that each patient in an intervention group has some probability of being assigned to all other intervention groups as well, it is important that the distributions overlap. This overlap was graphically checked. In case of serious non-overlap, some covariate(s) were omitted, and the PSs were recalculated.

*Balance check.* Each covariate was checked to determine whether their distribution was similar in the intervention groups. The balance before the PS corrections was checked by ANOVA, logistic regression, and multinomial logistic regression for continuous, dichotomous, and nominal variables. After correction, a balance check was done by ANCOVA with the intervention variable as a fixed factor and the multiple PSs as covariates for continuous variables. For each dichotomous variable, balance was checked by logistic regression with the variable as dependent, and the intervention and the PSs as independent variables. For a nominal variable, multinomial logistic regression was used with the intervention and PSs as independent variables. In supplementary tables, the p-values of these methods are shown before and after the correction. If, for a covariate, a balance was not achieved, that covariate was omitted from the estimation of the propensity score.

*Effect estimation after correction.* There are many approaches to using propensity scores: propensity score matching, weighting, stratification, adjustment with the propensity score, etc. In this study, most of the intervention variables were nominal and had several categories, and the multiple propensity score adjustment was used for all types of interventions. For dichotomous variables, the propensity score stratification into five strata was additionally used, and the adjustment was done with the quintiles. This paper only shows adjustments to the propensity score itself (but the results were very similar to those of quintiles). Finally, the RRs of postoperative pulmonary complications (PPC) were calculated using a generalised linear model for repeated measures design using Poisson distribution for the dependent variable with log link and with the adjustment of the multiple propensity scores.

**References**

1 Rosenbaum PR, Rubin DB. The central role of the propensity score in observational studies for causal effects. *Biometrika* 1983; **70**: 41-55

2 Imbens G. The role of the propensity score in estimating dose-response functions. *Biometrika* 2000; **87**: 706-10

**Supplemental Table legend**

**Supplemental Table 1S.** Covariates *used (x)* or not *used (-)* in the propensity score (PS) models for intraoperative respiratory critical events

**Supplemental Table 2S.** Descriptive statistics* of the covariates in the *American Society of Anaesthesiologists (ASA) physical status* groups (ASA <3 vs ASA≥3) and p-values for the differences before and after adjustment with propensity score (PS)

**Supplemental Table 3S.** Descriptive statistics* of the covariates in the *ventilation strategy* groups and p-values for the differences before and after adjustment with propensity score (PS)

**Supplemental Table 4S.** Descriptive statistics* of the covariates in the *airway management* groups (facemask, tracheal tube, supraglottic airway) and p-values for the differences before and after adjustment with propensity score (PS)

**Supplemental Table 5S.** Descriptive statistics* of the covariates in the *Neuromuscular blocking agent (NMBA)* used groups and p-values for the differences before and after adjustment with propensity score (PS)

**Supplemental Table 6S.** Descriptive statistics* of the covariates in the *Current respiratory and airway problems* groups and p-values for the differences before and after adjustment with propensity score (PS)

**Supplemental Table 7S.** Descriptive statistics* of the covariates in the *Breathing condition on the day of anaesthesia groups and p-values for the differences before and after adjustment with propensity sc*ore (PS).

**Supplemental Table 8S.** Comparison between open and non-invasive surgery in the incidence of intraoperative intervention for hypoxaemia and/or hypo- or hypercapnia and postoperative pulmonary complications (PPC) at 30 days. Data are corrected for age and weight.

**Supplemental Table 9S.** Interventions performed in the 820 patients who experienced intraoperative hypoxemia. Results express potential multiple interventions.

**Supplemental Table 1S.** Covariates *used (x)* or not *used (-)* in the propensity score (PS) models for intraoperative respiratory critical events

| Covariate | ASA >=3 | Ventilation strategy | Airway management | NMBA used | Current respiratory and airway problems | Breathing condition at day of anaesthesia |
| --- | --- | --- | --- | --- | --- | --- |
| Sex | x | x | x | x | x |  |
| Corrected age | x | x | x | x | x | x |
| Weight | x | x | x | x | x | x |
| Type of procedure  (surgical vs not surgical) | x | x | x | x | x | x |
| Type of surgery |  |  |  |  |  |  |
| Gastro-intestinal surgery | x | x | x | x | x | x |
| Thoracic surgery | x | x |  | x | x | - |
| Cardiac surgery | x | x |  | x | x | x |
| Genitourinary surgery | x | x | x | x | x | - |
| Neurosurgery | x | - | x | x | - | x |
| Opthalmology surgery | x | x | x | x | x | - |
| ENT-Plastic surgery | x | x | x | x | x | - |
| Orthopaedic surgery | x | x | x | x | x | - |
| Dermatology surgery | x | x | x | x | x | - |
| Current Respiratory and Airway Problem on the day of anaesthesia | - | x | x | x | x | x |
| Breathing condition on the day of anaesthesia | - | - | - | x | - | - |

**Supplemental Table 2S.** Descriptive statistics* of the covariates in the *American Society of Anaesthesiologists (ASA) physical status* groups (ASA <3 vs ASA≥3) and p-values for the differences before and after adjustment with propensity score (PS).

| Variable | ASA <3  n=3897 | ASA ≥3  n=2617 | Before PS correction (p) | After PS correction (p) |
| --- | --- | --- | --- | --- |
| Sex (male) | 68.2% | 59.3% | <0.001 | 0.932 |
| Corrected age | 46.59 (6.3) | 42.00 (7.38) | <0.001 | 0.734 |
| Weight | 4.60 (1.4) | 3.36 (1.3) | <0.001 | 0.597 |
| Type of procedure (surgical) | 82.3% | 75.2% | <0.001 | 0.934 |
| Gastro-intestinal surgery | 53.8% | 42.3% | <0.001 | 0.918 |
| Thoracic surgery | 0.4% | 1.7% | <0.001 | 0.975 |
| Cardiac surgery | 0.6% | 15.7% | <0.001 | 0.173 |
| Genitourinary surgery | 7.2% | 2.6% | <0.001 | 0.976 |
| Neurosurgery | 3.7% | 7.2% | <0.001 | 0.941 |
| Opthalmology surgery | 2.4% | 1.7% | 0.047 | 0.987 |
| ENT-Plastic surgery | 6.5% | 3.3% | <0.001 | 0.982 |
| Orthopaedic surgery | 4.8% | 0.6% | <0.001 | 0.625 |
| Dermatology surgery | 3.3% | 1.0% | <0.001 | 0.952 |

*For categorical variables percentages, for continuous variables mean(SD)

**Supplemental Table 3S.** Descriptive statistics* of the covariates in the *ventilation strategy* groups and p-values for the differences before and after adjustment with propensity score (PS).

| Variable | Spontaneous without airway managament n=947 | Spontaneous with airway managent  n=383 | Assisted  n=516 | Volume controlled (VC)  n=762 | Pressure controlled (PC)  n=3414 | Pressure regulated volume controlled (PRVC)  n=457 | Before PS correction (p) | After PS correction (p) |
| --- | --- | --- | --- | --- | --- | --- | --- | --- |
| Sex (male) | 68.2% | 71.0% | 68.0% | 62.7% | 62.9% | 64.8% | <0.001 | 0.99 |
| Corrected age | 45.72 (6.06) | 47.81 (5.96) | 46.86 (6.41) | 45.31 (6.78) | 43.83 (7.41) | 44.68 (7.02) | <0.001 | 0.733 |
| Weight | 4.35 (1.39) | 4.81 (1.48) | 4.47 (1.46) | 4.13 (1.44) | 3.91 (1.53) | 4.16 (1.51) | <0.001 | 0.925 |
| Type of procedure (surgical) | 59.1% | 60.6% | 71.9% | 86.5% | 85.5% | 87.3% | <0.001 | 0.14 |
| Gastro-intestinal surgery | 42.3%/ | 40.2% | 47.1% | 53.0% | 51.1% | 52.5% | <0.001 | 0.848 |
| Thoracic surgery | 0.2% | 0.3% | 0.4% | 1.3% | 1.0% | 2.2% | <0.001 | 0.511 |
| Cardiac surgery | 0.1% | 0.3% | 0.8% | 9.1% | 8.8% | 10.5% | 0.002 | 0.06 |
| Genitourinary surgery | 3.0% | 4.7% | 6.8% | 6.3% | 5.4% | 7.4% | <0.001 | 0.995 |
| Neurosurgery | 0.1% | 0.0% | 0.8% | 7.2% | 6.6% | 5.1% | <0.001 | 0.002 |
| Ophthalmology surgery | 0.7% | 0.5% | 3.1% | 1.4% | 2.7% | 2.8% | <0.001 | 0.994 |
| ENT-Plastic surgery | 4.0% | 2.9% | 4.3% | 5.5% | 5.9% | 4.5% | 0.008 | 0.8 |
| Orthopaedic surgery | 5.4% | 8.1% | 4.7% | 2.2% | 2.1% | 1.8% | <0.001 | 0.24 |
| Dermatology surgery | 3.4% | 3.9% | 4.8% | 1.0% | 1.9% | 1.8% | <0.001 | 0.905 |
| Current Respiratory and Airway Problem on the day of anaesthesia | 17.0% | 15.9% | 20.2% | 14.7% | 17.5% | 18.0% | 0.206 | 0.467 |

*For categorical variables percentages, for continuous variables mean(SD)

**Supplemental Table 4S.** Descriptive statistics* of the covariates in the *airway management* groups (facemask, tracheal tube, supraglottic airway) and p-values for the differences before and after adjustment with propensity score (PS).

| Variable | Facemask n=727 | Tracheal tube n=4665 | Supraglottic Airway  n=720 | Before PS correction (p) | After PS correction (p) |
| --- | --- | --- | --- | --- | --- |
| Sex (male) | 65.7% | 62.%) | 71% | <0.001 | 0.985 |
| Corrected age | 46.56 (6.02) | 43.94 (7.36) | 47.99 (5.77) | <0.001 | 0.671 |
| Weight | 4.56 (1.41) | 3.91 (1.52) | 4.92 (1.40) | <0.001 | 0.503 |
| Type of procedure (surgical) | 56.5% | 85.5% | 71.4% | <0.001 | 0.984 |
| Gastro-intestinal surgery | 34.3% | 51.6% | 46.1% | <0.001 | 0.918 |
| Thoracic surgery | 0.1% | 1.2% | 0.1% | 0.012 | 0.98 |
| Cardiac surgery | 0.1% | 9.3% | 0% | 0.049 | 0.769 |
| Genitourinary surgery | 3.7% | 5.7% | 6.5% | <0.001 | 0.149 |
| Neurosurgery | 0.1% | 6.9% | 5.3% | <0.001 | 0.922 |
| Ophthalmology surgery | 1% | 2.3% | 3.2% | 0.019 | 0.084 |
| ENT-Plastic surgery | 4.0% | 6.0% | 0.8% | 0.007 | 0.079 |
| Orthopaedic surgery | 8.3% | 1.5% | 9.9% | <0.001 | 0.222 |
| Dermatology surgery | 5.4% | 1.7% | 4.7% | <0.001 | 0.921 |
| Current Respiratory and Airway Problem on the day of anaesthesia | 15.7% | 19.2% | 9.3% | <0.001 | 0.875 |

*For categorical variables percentages, for continuous variables mean(SD)

**Supplemental Table 5S.** Descriptive statistics* of the covariates in the *Neuromuscular blocking agent (NMBA)* used groups and p-values for the differences before and after adjustment with propensity score (PS).

| Variable | No NMBA n=2669 | NMBA prior induction  n=2831 | NMBA after induction  n=801 | Before PS correction (p) | After PS correction (p) |
| --- | --- | --- | --- | --- | --- |
| Sex (male) | 65.2% | 64.7% | 59.2% | 0.007 | 0.99 |
| Corrected age | 46.46 (6.53) | 44.65 (6.77) | 39.70 (8.22) | <0.001 | 0.982 |
| Weight | 4.49 (1.47) | 4.09 (1.42) | 2.91 (1.46) | <0.001 | 0.977 |
| Type of procedure (surgical) | 65.9% | 87.6% | 90.6% | <0.001 | 0.655 |
| Gastro-intestinal surgery | 38.9% | 53.0% | 58.6% | <0.001 | 0.904 |
| Thoracic surgery | 0.5% | 1.0% | 2.0% | <0.001 | 0.992 |
| Cardiac surgery | 0.6% | 10.2% | 16.1% | <0.001 | 0.342 |
| Genitourinary surgery | 5.4% | 6.3% | 2.4% | 0.06 | 0.935 |
| Neurosurgery | 2.6% | 7.6% | 5.9% | <0.001 | 0.708 |
| Ophthalmology surgery | 2.0% | 2.5% | 1.7% | 0.882 | 0.967 |
| ENT-Plastic surgery | 7.0% | 4.2% | 4.0% | <0.001 | 0.978 |
| Orthopaedic surgery | 5.7% | 1.6% | 0.4% | <0.001 | 0.964 |
| Dermatology surgery | 3.7% | 1.7% | 0.9% | <0.001 | 0.964 |
| Current Respiratory and Airway Problem on the day of anaesthesia | 17.3% | 13.6% | 39.3% | <0.001 | 0.945 |
| Breathing condition on the day of anaesthesia |  |  |  | <0.001 | 0.997 |
| No oxygen (O_2_), no ventilation assistance | 86.2% | 82.4% | 26.4% |  |  |
| Patient under spontaneous ventilation with O_2_ | 5.0% | 8.1% | 4.6% |  |  |
| Patient under NIV with CPAP | 2.2% | 3.6% | 1.4% |  |  |
| Patient INTUBATED on conventional ventilation | 6.3% | 5.7% | 59.9% |  |  |
| Patient INTUBATED on HFOV | 0.3% | 0.1% | 5.9% |  |  |
| Patient on ECMO | 0.1% | 0.1% | 1.9% |  |  |

*For categorical variables percentages, for continuous variables mean(SD)

Abbreviations: CPAP=continous positive airway pressure, NIV=non-invasive ventilation, HFOV=high-frequency oscillatory ventilation, ECMO=extracorporal membrane oxygenation

**Supplemental Table 6S.** Descriptive statistics* of the covariates in the *Current respiratory and airway problems* groups and p-values for the differences before and after adjustment with propensity score (PS).

| Variable | No  n=5327 | Yes  n=1192 | Before PS correction (p) | After PS correction (p) |
| --- | --- | --- | --- | --- |
| Sex (male) | 65.3% | 61.5% | 0.013 | 0.994 |
| Corrected age | 45.32 (6.75) | 42.19 (8.17) | <0.001 | 0.97 |
| Weight | 4.28 (0.47) | 3.29 (1.50) | <0.001 | 0.953 |
| Type of procedure (surgical) | 81.8% | 68.6% | <0.001 | 0.974 |
| Gastro-intestinal surgery | 51.3% | 39.8% | <0.001 | 0.983 |
| Thoracic surgery | 0.6% | 2.3% | <0.001 | 0.981 |
| Cardiac surgery | 6.3% | 8.1% | 0.033 | 0.995 |
| Genitourinary surgery | 6.3% | 0.9% | <0.001 | 0.77 |
| Neurosurgery | 5.1% | 4.8% | 0.607 | - |
| Ophthalmology surgery | 1.9% | 3.4% | <0.001 | 0.991 |
| ENT-Plastic surgery | 4.4% | 8.6% | <0.001 | 0.984 |
| Orthopaedic surgery | 3.7% | 0.7% | <0.001 | 0.862 |
| Dermatology surgery | 2.7% | 0.8% | <0.001 | 0.992 |
| Breathing condition on the day of anaesthesia |  |  | <0.001 | 0.937 |
| No oxygen (O_2_), no ventilation assistance | 87.5% | 33.0% |  |  |
| Patient under spontaneous ventilation with O_2_ | 3.4% | 19.2% |  |  |
| Patient under NIV with CPAP | 0.8% | 10.7% |  |  |
| Patient INTUBATED on conventional ventilation | 7.7% | 33.4% |  |  |
| Patient INTUBATED on HFOV | 0.3% | 3.6% |  |  |
| Patient on ECMO | 0.3% | 0.2% |  |  |

*For categorical variables percentages, for continuous variables mean(SD)

Abbreviations: CPAP=continous positive airway pressure, NIV=non-invasive ventilation, HFOV=high-frequency oscillatory ventilation, ECMO=extracorporal membrane oxygenation

**Supplemental Table 7S.** Descriptive statistics* of the covariates in the *Breathing condition on the day of anaesthesia groups and p-values for the differences before and after adjustment with propensity sc*ore (PS).

| Variable | No oxygen, no ventilation assistance n=5056 | Patient under spontaneous ventilation with O_2_ n=409 | Patient under NIV with CPAP n=173 | Patient INTUBATED on conventional ventilation n=811 | Patient INTUBATED on HFOV n=58 | Before PS correction (p) | After PS correction (p) |
| --- | --- | --- | --- | --- | --- | --- | --- |
| Sex (male) | 66.3% | 63.6% | 61.3% | 57.0% | 53.4% | <0.001 | 0.986 |
| Corrected age | 46.24 (6.36) | 42.28 (6.27) | 41.42 (7.18) | 38.28 (6.95) | 32.33 (7.79) | <0.001 | 0.077 |
| Weight | 4.46 (1.41) | 3.28 (1.09) | 3.08 (1.23) | 2.71 (1.21) | 1.46 (0.99) | <0.001 | 0.58 |
| Type of procedure (surgical) | 79.8% | 77.3% | 65.9% | 80.4% | 93.1% | <0.001 | 0.848 |
| Gastro-intestinal surgery | 50.7% | 46.0% | 27.2% | 45.9% | 65.5% | <0.001 | 0.854 |
| Thoracic surgery | 0.4% | 2.0% | 1.7% | 3.0% | 0.0% |  | - |
| Cardiac surgery | 4.1% | 10.5% | 16.2% | 16.8% | 22.4% | <0.001 | 0.914 |
| Genitourinary surgery | 6.4% | 1.7% | 0.0% | 2.1% | 0.0% |  | - |
| Neurosurgery | 4.6% | 8.8% | 5.2% | 5.9% | 5.2% | 0.006 | 0.886 |
| Ophthalmology surgery | 2.0% | 4.4% | 6.9% | 1.1% | 0.0% |  | - |
| ENT-Plastic surgery | 5.3% | 2.2% | 8.7% | 5.9% | 0.0% |  | - |
| Orthopaedic surgery | 3.9% | 0.7% | 0.6% | 0.5% | 0.0% |  | - |
| Dermatology surgery | 2.8% | 1.0% | 0.6% | 0.9% | 0.0% |  | - |
| Current Respiratory and Airway Problem on the day of anaesthesia | 7.8% | 56% | 73.8% | 49.2% | 74.1% | <0.001 | 0.162 |

*For categorical variables percentages, for continuous variables mean(SD)

**Supplemental Table 8S.** Comparison between open and non-invasive surgery in the incidence of intraoperative intervention for hypoxaemia and/or hypo- or hypercapnia and postoperative pulmonary complications (PPC) at 30 days. Data are adjusted for corrected age and weight.

|  |  | Open surgery | Minimally invasive surgery | RR (95% CI) | |
| --- | --- | --- | --- | --- | --- |
| Oesophageal, gastro-intestinal surgery | | | | |  |
| Hypoxaemia | No  Yes | 2593 (88.1%)  349 (11.9%) | 226 (84.6%)  41 (15.4%) | **1.50 (1.12 – 2.02)** | |
| Hypo-Hypercapnia | No  Yes | 2714 (92.3%)  228 (7.7%) | 222 (83.1%)  45 (16.9%) | **2.40 (1.79-3.23)** | |
| PPC at 30 days | No  Yes | 2180 (92.3%)  182 (7.7%) | 208 (94.5%)  12 (5.5%) | 0.54 (0.27-1.23) | |
| Thoracic surgery | | | | |  |
| Hypoxaemia | No  Yes | 32 (74.4%)  11 (25.6%) | 7 (46.7%)  8 (53.3%) | **2.13 (1.06-4.29)** | |
| Hypo-Hypercapnia | No  Yes | 30 (69.8%)  13 (30.2%) | 8 (53.3%)  7 (46.7%) | 1.51 (0.75-3.03) | |
| PPC at 30 days | No  Yes | 24 (66.7%)  12 (33.3%) | 9 (69.2%)  4 (30.8%) | 1.03 (0.40-2.68) | |
| Genitourinary surgery | | | | |  |
| Hypoxaemia | No  Yes | 254 (90.4%)  27 (9.6%) | 63 (91.3%)  6 (8.7%) | 0.61 (0.28-1.35) | |
| Hypo-Hypercapnia | No  Yes | 262 (93.2%)  19 (6.8%) | 66 (95.7%)  3 (4.3%) | 0.65 (0.16-2.64) | |
| PPC at 30 days | No  Yes | 223 (98.7%)  3 (1.3%) | 62 (98.4%)  1 (1.6%) | 1.11 (0.11-11.33) | |

Data are given in numbers (percentage) or relative risk (RR) with the corresponding 95% confidence interval (95CI)

**Supplemental Table 9S.** Interventions performed in the 820 patients who experienced intraoperative hypoxemia. Results express potential multiple interventions.

|  | No NMBA | NMBA prior to intubation | NMBA following intubation |
| --- | --- | --- | --- |
| Emergency/unplanned intubation | 31 (11.2) | 27 (7.2) | 3 (1.8) |
| Change/repositioning accidentally dislocated or obstructed TT | 34 (12.3) | 65 (17.3) | 28 (16.7) |
| Need FiO2 higher than for routine (or persistent FiO2 100%) | 120 (43.5) | 178 (47.3) | 108 (64.3) |
| Need for PEEP higher than for routine practice | 46 (16.7) | 47 (12.5) | 16 (9.5) |
| Need for prolonged manual ventilation in already intubated/ventilated patient | 58 (21) | 82 (21.8) | 53 (31.5) |
| Recruiting maneuver | 55 (19.9) | 111 (29.5) | 60 (35.7) |
| Switch from conventional to HFOV | 1 (0.4) | 2 (0.5) | 6 (3.6) |
| Drainage of acute pneumothorax | 1 (0.4) | 1(0.3) | 1 (0.6) |
| Pharmacological treatment of laryngospasm | 32 (11.6) | 27 (7.2) | 6 (3.6) |
| Pharmacological treatment of bronchospasm | 22 (8) | 11 (2.9) | 5 (3) |
| Other interventions | 50 (18.1) | 56 (14.9) | 123 (15) |
