# Supplementary material for: Ventilation strategies and risk factors for intraoperative respiratory critical events and postoperative pulmonary complications in neonates and small infants: a secondary analysis of the NECTARINE cohort☆
Source: Br J Anaesth. 2025 Feb 20;135(5):1528–36. doi: 10.1016/j.bja.2024.12.038 (PMC12597469; doi:10.1016/j.bja.2024.12.038)
Supplement: Multimedia component 2 [file mmc2.docx]

**NECTARINE Steering Committee**

Nicola Disma^1^

Francis Veyckemans^2^

Katalin Virag^3^

Tom G. Hansen^4^

Karin Becke-Jakob^5^

Pierre Harlet^6^

Laszlo Vutskits^7^

Suellen M. Walker^8^

Jurgen C. de Graaff^9^

Marzena Zielinska^10^

Dusica Simic^11^

Thomas Engelhardt^12^

Walid Habre^7^

1 Department of Anaesthesia, Unit for Research & Innovation, Istituto Giannina Gaslini, Genova, Italy

2 Département d'Anaesthésie-Réanimation pédiatrique, Hôpital Jeanne de Flandre, CHRU de Lille, Lille, France

3 Department of Medical Physics and Informatics, University of Szeged, Szeged, Hungary

4 Department of Anaesthesia and Intensive Care - Paediatrics, Odense University Hospital, Odense, Denmark; Department of Clinical Research - Anaesthesiology, University of Southern Denmark, Odense, Denmark

5 Department of Anaesthesia and Intensive Care, Cnopf Children's Hospital/Hospital Hallerwiese, Nürnberg, Germany

6 Research Department, European Society of Anaesthesiology, Brussels, Belgium

7 Department of Anaesthesiology, Pharmacology, Intensive Care and Emergency Medicine, University Hospitals of Geneva, Geneva, Switzerland; University of Geneva, Geneva, Switzerland

8 Department of Paediatric Anaesthesia, Great Ormond St Hospital NHS Foundation Trust, London, UK

9 Department of Anesthesia, Erasmus MC- Sophia Children's Hospital, Rotterdam, The Netherlands

10 Department of Paediatric Anaesthesiology and Intensive Care, Wroclaw Medical University, Wroclaw, Poland

11 Department of Pediatric Anesthesia and Intensive Care, University Children's Hospital, Medical Faculty University of Belgrade, Belgrade, Serbia

12 Department of Anaesthesia, Montreal Children's Hospital, Montreal, QC, Canada
