# Supplementary material for: Ventilation strategies and risk factors for intraoperative respiratory critical events and postoperative pulmonary complications in neonates and small infants: a secondary analysis of the NECTARINE cohort☆
Source: Br J Anaesth. 2025 Feb 20;135(5):1528–36. doi: 10.1016/j.bja.2024.12.038 (PMC12597469; doi:10.1016/j.bja.2024.12.038)
Supplement: Multimedia component 3 [file mmc3.docx]

Each NECTARINE collaborator with the format:

First name LAST NAME, Hospital, COUNTRY

**AUSTRIA**
• Christian BRESCHAN, Klinikum Klagenfurt, AUSTRIA
• Rudolf LIKAR, Klinikum Klagenfurt, AUSTRIA (Site Local Investigator)
• Manuela PLATZER, Klinikum Klagenfurt, AUSTRIA
• Isole EDELMAN, Medical University Graz, AUSTRIA
• Johanes EGER, Medical University Graz, AUSTRIA
• Stefan HESCHL, Medical University Graz, AUSTRIA
• Brigitte MESSERER, Medical University Graz, AUSTRIA
• Maria VITTINGHOFF, Medical University Graz, AUSTRIA (National Coordinator, Site Local Investigator)
• Ruth KROESS, University Innsbruck, AUSTRIA (Site Local Investigator)
• Martina STICHLBERGER, University Innsbruck, AUSTRIA

**BELGIUM**
• David KAHN, Cliniques universitaires St Luc, BELGIUM (Site Local Investigator)
• Thierry PIROTTE, Cliniques universitaires St Luc, BELGIUM
• Caroline PREGARDIEN, Cliniques universitaires St Luc, BELGIUM
• Francis VEYCKEMANS, Cliniques universitaires St Luc, BELGIUM (National Coordinator)
• France STEVENS, Grand Hôpital de Charleroi, BELGIUM (Site Local Investigator)
• Johan BERGHMANS, Queen Paola Children's Hospital, BELGIUM (Site Local Investigator)
• Annemie BAUTERS, University Hospital Ghent, BELGIUM
• Luc DE BAERDEMAEKER, University Hospital Ghent, BELGIUM
• Stefan DE HERT, University Hospital Ghent, BELGIUM (Site Local Investigator)
• Koen LAPAGE, University Hospital Ghent, BELGIUM
• Aliaksandra PARASHCHANKA, University Hospital Ghent, BELGIUM
• Jurgen VAN LIMMEN, University Hospital Ghent, BELGIUM
• Piet WYFFELS, University Hospital Ghent, BELGIUM
• Julie LAUWERYNS, University hospitals Leuven, BELGIUM (Site Local Investigator)
• Nadia NAJAFI, Universitair Ziekenhuis Brussel (UZ Brussel), BELGIUM (Site Local Investigator)
• Joris VUNDELINCKX, Ziekenhuis Oost-Limburg, BELGIUM (Site Local Investigator)

**CROATIA**
• Diana BUTKOVIĆ, Children's Hospital Zagreb, CROATIA
• Ivana KEROVEC SORIĆ, Children's Hospital Zagreb, CROATIA
• Sandra KRALIK, Children's Hospital Zagreb, CROATIA (National Coordinator, Site Local Investigator)
• Ana MARKIĆ, Children's Hospital Zagreb, CROATIA
• Josip AZMAN, Rijeka University Hospital, CROATIA (Site Local Investigator)
• Josko MARKIC, University hospital Split, CROATIA
• Daniela PUPACIC, University hospital Split, CROATIA (Site Local Investigator)

**CZECH REPUBLIC**
• Michal FRELICH, Faculty Hospital Ostrava, CZECH REPUBLIC
• Petr REIMER, Faculty Hospital Ostrava, CZECH REPUBLIC
• René URBANEC, Faculty Hospital Ostrava, CZECH REPUBLIC (Site Local Investigator)
• Petra CAJKOVÁ, Fakultni Nemocnice Motol, CZECH REPUBLIC
• Vladimír MIXA, Fakultni Nemocnice Motol, CZECH REPUBLIC (Site Local Investigator)
• Yvona SEDLÁČKOVÁ, University Hospital Brno, CZECH REPUBLIC
• Lenka KNOPPOVÁ, University Hospital Brno, CZECH REPUBLIC
• Alena ZLÁMALOVÁ (NEÉ KVĚTOŇOVÁ), University Hospital Brno, CZECH REPUBLIC
• Martin VAVŘINA, University Hospital Brno, CZECH REPUBLIC (Site Local Investigator)
• Jiří ŽUREK, University Hospital Brno, CZECH REPUBLIC (National Coordinator)

**DENMARK**
• Tom HANSEN, Odense University Hospital, DENMARK (National Coordinator, Site Local Investigator)
• Arash AFSHARI, Rigshospitalet, Department of Anesthesia, Juliane Marie Center – University of Copenhagen, DENMARK (Site Local Investigator)
• Anders BASTHOLM BILLE, Rigshospitalet, Department of Anesthesia, Juliane Marie Center – University of Copenhagen, DENMARK
• Marguerite ELLEKVIST, Rigshospitalet, Department of Anesthesia, Juliane Marie Center – University of Copenhagen, DENMARK

**ESTONIA**
• Mari-Liis ILMOJA, Tallinn Children’s Hospital, ESTONIA (Site Local Investigator)
• Reet MOOR, Tallinn Children’s Hospital, ESTONIA
• Reet KIKAS, Tartu University Hospital, ESTONIA (Site Local Investigator, National Coordinator)
• Merle VÄLI, Tartu University Hospital, ESTONIA

**FINLAND**
• Kariantti KALLIO, Helsinki University Central Hospital, FINLAND
• Elisa REPONEN, Helsinki University Central Hospital, FINLAND
• Pertti SUOMINEN, Helsinki University Central Hospital, FINLAND
• Sami SUVANTO, Helsinki University Central Hospital, FINLAND (Site Local Investigator)
• Raisa VÄHÄTALO, Helsinki University Central Hospital, FINLAND
• Hannu KOKKI, Kuopio University Hospital, FINLAND (Site Local Investigator)
• Merja KOKKI, Kuopio University Hospital, FINLAND
• Jarkko HARJU, Tampere University Hospital, FINLAND
• Miia KOKKONEN, Tampere University Hospital, FINLAND
• Jenni VIERI, Tampere University Hospital, FINLAND (Site Local Investigator)
• Tuula MANNER, Turku University Hospital, FINLAND (Site Local Investigator, National Coordinator)

**FRANCE**
• Catherine AMORY, American Memorial Hospital CHU Reims, FRANCE
• Hugues LUDOT, American Memorial Hospital CHU Reims, FRANCE (Site Local Investigator)
• Dina BERT, CHRU de Lille Hôpital Jeanne de Flandre, FRANCE
• Juliette GODART, CHRU de Lille Hôpital Jeanne de Flandre, FRANCE
• Anne LAFFARGUE, CHRU de Lille Hôpital Jeanne de Flandre, FRANCE (Site Local Investigator, National Co‐Coordinator)
• Hervé DUPONT, CHU Amiens Picardie, FRANCE (Site Local Investigator)
• Benjamin URBINA, CHU Amiens Picardie, FRANCE
• Catherine BAUJARD, CHU Bicetre, FRANCE (Site Local Investigator)
• Philippe ROULLEAU, CHU Bicetre, FRANCE
• Giuseppe STAITI, CHU Bicetre, FRANCE
• Maryline BORDES, CHU de Bordeaux, FRANCE
• Karine NOUETTE GAULAIN, CHU de Bordeaux, FRANCE (Site Local Investigator)
• Yann HAMONIC, CHU de Bordeaux, FRANCE
• François SEMJEN, CHU de Bordeaux, FRANCE
• Olivier JAQMARQC, CHU de Nantes, FRANCE
• Caroline LEJUS-BOURDEAU, CHU de Nantes, FRANCE
• Cécile MAGNE, CHU de Nantes, FRANCE (Site Local Investigator)
• Léa PETRY, CHU Nancy, FRANCE
• Lilica ROS, CHU Nancy, FRANCE
• Aurélien ZANG, CHU Nancy, FRANCE (Site Local Investigator)
• Mehdi BENNIS, CHU Toulouse, FRANCE (Site Local Investigator)
• Bernard COUSTETS, CHU Toulouse, FRANCE (Site Local Investigator)
• Rose FESSEAU, CHU Toulouse, FRANCE
• Isabelle CONSTANT, Hopital Armand Trousseau, FRANCE
• Eliane KHALIL, Hopital Armand Trousseau, FRANCE (Site Local Investigator)
• Nada SABOURDIN, Hopital Armand Trousseau, FRANCE
• Noemie AUDREN, Hopital couple enfant CHU de Grenoble, FRANCE (Site Local Investigator)
• Thomas DESCARPENTRIES, Hopital couple enfant CHU de Grenoble, FRANCE
• Fanny FABRE, Hopital couple enfant CHU de Grenoble, FRANCE
• Aurélien LEGRAND, Hopital couple enfant CHU de Grenoble, FRANCE
• Emilie DRUOT, Hôpital Universitaire Necker Enfants Malades, FRANCE
• Gilles ORLIAGUET, Hôpital Universitaire Necker Enfants Malades, FRANCE (Site Local Investigator)
• Lucie SABAU, Hôpital Universitaire Necker Enfants Malades, FRANCE
• Lynn UHRIG, Hôpital Universitaire Necker Enfants Malades, FRANCE
• François DE LA BRIÈRE, Hôpitaux Pédiatriques de Nice CHU Lenval, FRANCE (Site Local Investigator)
• Karin JONCKHEER, Hôpitaux Pédiatriques de Nice CHU Lenval, FRANCE
• Jean-Paul MISSION, Hôpitaux Pédiatriques de Nice CHU Lenval, FRANCE
• Lucia SCORDO, Hôpitaux Pédiatriques de Nice CHU Lenval, FRANCE
• Caroline COUCHEPIN, Lapeyronie – CHU of Montpellier, FRANCE
• Christophe DADURE, Lapeyronie – CHU of Montpellier, FRANCE (Site Local Investigator, National Co‐Coordinator)
• Pablo DE LA ARENA, Lapeyronie – CHU of Montpellier, FRANCE
• Laurent HERTZ, Lapeyronie – CHU of Montpellier, FRANCE
• Philippe PIRAT, Lapeyronie – CHU of Montpellier, FRANCE
• Chrystelle SOLA, Lapeyronie – CHU of Montpellier, FRANCE
• Myriam BELLON, Robert Debré University Hospital, FRANCE
• Souhayl DAHMANI, Robert Debré University Hospital, FRANCE (Site Local Investigator)
• Florence JULIEN-MARSOLLIER, Robert Debré University Hospital, FRANCE
• Daphne MICHELET, Robert Debré University Hospital, FRANCE
• Veronique DEPRET-DONATIEN, Teaching Hospital of Caen, FRANCE (Site Local Investigator)
• Anne LESAGE, Teaching Hospital of Caen, FRANCE

**GERMANY**
• Jost KAUFMANN, Children’s Hospital Cologne, GERMANY
• Michael LASCHAT, Children’s Hospital Cologne, GERMANY (Site Local Investigator)
• Frank WAPPLER, Children’s Hospital Cologne, GERMANY
• Karin BECKE, Cnopf Children’s Hospital, GERMANY (Site Local Investigator, National Coordinator)
• Lena BRUNNER, Cnopf Children’s Hospital, GERMANY
• Karin OPPENRIEDER, Cnopf Children’s Hospital, GERMANY
• Gregor BADEL, Klinik St. Hedwig, GERMANY
• Karin HOCHMUTH, Klinik St. Hedwig, GERMANY
• Bernhard KOLLER, Klinik St. Hedwig, GERMANY
• Anita REIL, Klinik St. Hedwig, GERMANY
• Sebastian RICHTER, Klinik St. Hedwig, GERMANY (Site Local Investigator)
• Thomas FISCHER, Klinikum Kassel, GERMANY (Site Local Investigator)
• Anja DIERS, Klinikum Oldenburg, med. Campus der Universität, GERMANY
• Clemens SCHORER, Klinikum Oldenburg, med. Campus der Universität, GERMANY
• Andreas WEYLAND, Klinikum Oldenburg, med. Campus der Universität, GERMANY (Site Local Investigator)
• Ruth COHAUSZ, Klinikum Stuttgart Olgahospital, GERMANY
• Franz-Josef KRETZ, Klinikum Stuttgart Olgahospital, GERMANY (Site Local Investigator)
• Michaela LÖFFLER, Klinikum Stuttgart Olgahospital, GERMANY
• Markus WILBS, Klinikum Stuttgart Olgahospital, GERMANY
• Claudia HOEHNE, Universitaetsklinikum Leipzig, GERMANY (Site Local Investigator, National Coordinator)
• Johanna ULRICI, Universitaetsklinikum Leipzig, GERMANY
• Christiane GOETERS, University Hospital Münster, GERMANY (Site Local Investigator)
• Armin FLINSPACH, University Hospital Frankfurt/Main, GERMANY
• Matthias KLAGES, University Hospital Frankfurt/Main, GERMANY (Site Local Investigator)
• Simone LINDAU, University Hospital Frankfurt/Main, GERMANY
• Leila MESSROGHLI, University Hospital Frankfurt/Main, GERMANY
• Kai ZACHAROOWSKI, University Hospital Frankfurt/Main, GERMANY
• Christoph EISNER, University Hospital Heidelberg, GERMANY
• Thomas MUELLER, University Hospital Heidelberg, GERMANY (Site Local Investigator)
• Daniel RICHTER, University Hospital Heidelberg, GERMANY
• Melanie SCHÄFER, University Hospital Heidelberg, GERMANY
• Markus WEIGAND, University Hospital Heidelberg, GERMANY
• Sebastian WEITERER, University Hospital Heidelberg, GERMANY
• Miriam OCHSENREITER, University Medical Centre Mannheim, GERMANY
• Michael SCHÖLER, University Medical Centre Mannheim, GERMANY (Site Local Investigator)
• Tom TERBOVEN, University Medical Centre Mannheim, GERMANY
• Isabel EGGEMANN, University Hospital of Cologne, GERMANY
• Sascha HAUSSMANN, University Hospital of Cologne, GERMANY
• Nicolas LEISTER, University Hospital of Cologne, GERMANY
• Christoph MENZEL, University Hospital of Cologne, GERMANY
• Uwe TRIESCHMANN, University Hospital of Cologne, GERMANY
• Sirin YÜCETEPE, University Hospital of Cologne, GERMANY (Site Local Investigator)
• Susanna KEILIG, University of Wuerzburg, GERMANY
• Peter KRANKE, University of Wuerzburg, GERMANY (Site Local Investigator)
• Yvonne JELTING, University of Wuerzburg, GERMANY
• Torsten BAEHNER, University of Bonn, GERMANY
• Richard ELLERKMANN, University of Bonn, GERMANY (Site Local Investigator)
• Shahab GHAMARI, University of Bonn, GERMANY
• Claudia NEUMANN, University of Bonn, GERMANY
• Martin SÖHLE, University of Bonn, GERMANY

**GREECE**
• Pelagia CHLOROPOULOU, Democritus University of Thrace, GREECE (Site Local Investigator)
• Vagia NTRITSOU, "G. Gennimatas" General Hospital of Thessaloniki, GREECE (Site Local Investigator)
• Pinelopi PAPAGIANNOPOULOU, "G. Gennimatas" General Hospital of Thessaloniki, GREECE
• Eleana GARINI, General Pediatric Hospital "Agia Sophia", GREECE
• Afroditi KARAFOTIA, General Pediatric Hospital "Agia Sophia", GREECE
• Panagoula MAMMI, General Pediatric Hospital "Agia Sophia", GREECE (Site Local Investigator)
• Evangelia BALI, Hippokrateio General Hospital, Thessaloniki, GREECE
• Despoina IORDANIDOU, Hippokrateio General Hospital, Thessaloniki, GREECE (Site Local Investigator)
• Anna MALISIOVA, P & A Kyriakou Children Hospital, GREECE (National Coordinator, Site Local Investigator)
• Artemis POLYZOI, P & A Kyriakou Children Hospital, GREECE
• Adelais TSIOTOU, P & A Kyriakou Children Hospital, GREECE

**HUNGARY**
• Erzsebet SAPI, Gottsegen Hungarian Institute of Cardiology, HUNGARY (Site Local Investigator)
• Edgar SZÉKELY, Gottsegen Hungarian Institute of Cardiology, HUNGARY
• Nandor KOSIK, Heim Pal National Pediatric Institute, HUNGARY
• Veronika MARÁCZI, Heim Pal National Pediatric Institute, HUNGARY (Site Local Investigator)
• Janos SCHNUR, Heim Pal National Pediatric Institute, HUNGARY
• Judit CSILLAG, Semmelweis University, HUNGARY
• János GÁL, Semmelweis University, HUNGARY
• Gergely GÖBL, Semmelweis University, HUNGARY
• Balázs HAUSER, Semmelweis University, HUNGARY (Site Local Investigator)
• András PÉTROCY, Semmelweis University, HUNGARY
• Gyula TÖVISHÁZI, Semmelweis University, HUNGARY
• Andrea SZÉKELY, Semmelweis University, HUNGARY (National Coordinator)

**IRELAND**
• Stuart BLAIN, Children’s Health Ireland – Crumlin, IRELAND
• Sarah GALLAGHER, Children’s Health Ireland – Crumlin, IRELAND
• Sinead HARTE, Children’s Health Ireland – Crumlin, IRELAND
• Mandy JACKSON, Children’s Health Ireland – Crumlin, IRELAND
• Emma MEEHAN, Children’s Health Ireland – Crumlin, IRELAND
• Zeenat NAWOOR, Children’s Health Ireland – Crumlin, IRELAND
• Brendan O’HARE, Children’s Health Ireland – Crumlin, IRELAND (National Coordinator, Site Local Investigator)
• Mark ROSS, Children’s Health Ireland – Crumlin, IRELAND

**ITALY**
• Daniela LERRO, A.O.R.N Santobono Pausilipon, ITALY (Site Local Investigator)
• Marinella ASTUTO, A.O.U. Policlinico- Vittorio Emanuele Catania, ITALY (Site Local Investigator)
• Chiara GRASSO, A.O.U. Policlinico- Vittorio Emanuele Catania, ITALY
• Rita SCALISI, A.O.U. Policlinico- Vittorio Emanuele Catania, ITALY
• Giulia FRASACCO, AOU A. Meyer, ITALY
• Elena LENARES, AOU A. Meyer, ITALY (Site Local Investigator)
• Roberto LEONE, AOU A. Meyer, ITALY
• Maurizia GRAZZINI, Azienda Ospedaliera di Padova, ITALY
• Carmelo MINARDI, Azienda Ospedaliera di Padova, ITALY
• Nicola ZADRA, Azienda Ospedaliera di Padova, ITALY (Site Local Investigator)
• Gilda CINNELLA, Azienda Ospedaliero Universitaria Ospedali Riuniti Di Foggia, ITALY
• Antonella COTOIA, Azienda Ospedaliero Universitaria Ospedali Riuniti Di Foggia, ITALY
• Dario GALANTE, Azienda Ospedaliero Universitaria Ospedali Riuniti Di Foggia, ITALY (Site Local Investigator)
• Brita DE LORENZO, Azienda Ospedaliero-universitaria Pisana, ITALY
• Beate KUPPERS, Azienda Ospedaliero-universitaria Pisana, ITALY (Site Local Investigator)
• Giulia BOTTAZZI, Azienda ospedaliero-universitaria Policlinico S.Orsola Malpighi, ITALY
• Fabio CARAMELLI, Azienda ospedaliero-universitaria Policlinico S.Orsola Malpighi, ITALY
• Maria Cristina MONDARDINI, Azienda ospedaliero-universitaria Policlinico S.Orsola Malpighi, ITALY (Site Local Investigator)
• Emanuele ROSSETTI, Bambino Gesù Children's Hospital, IRCCS, ITALY
• Sergio PICARDO, Bambino Gesù Children's Hospital, IRCCS, ITALY
• Alessandro VITTORI, Bambino Gesù Children's Hospital, IRCCS, ITALY (Site Local Investigator)
• Anna CAMPORESI, Children Hospital Vittore Buzzi, ITALY (Site Local Investigator)
• Edoardo CALDERINI, Fondazione IRCCS Ca' Granda Ospedale Maggiore Policlinico, ITALY
• Laura Brigitta COLANTONIO, Fondazione IRCCS Ca' Granda Ospedale Maggiore Policlinico, ITALY
• Simona Anna FINAMORE, Fondazione IRCCS Ca' Granda Ospedale Maggiore Policlinico, ITALY (Site Local Investigator)
• Giuliana Anna PORRO, Fondazione IRCCS Ca' Granda Ospedale Maggiore Policlinico, ITALY
• Rachele BONFIGLIO, Istituto Giannina Gaslini, ITALY
• Nicola DISMA, Istituto Giannina Gaslini, ITALY (Site Local Investigator, National Coordinator)
• Svetlana KOTZEVA, Istituto Giannina Gaslini, ITALY
• Leila MAMELI, Istituto Giannina Gaslini, ITALY
• Girolamo MATTIOLI, Istituto Giannina Gaslini, ITALY
• Camilla MICALIZZI, Istituto Giannina Gaslini, ITALY
• Alessia MONTAGUTI, Istituto Giannina Gaslini, ITALY
• Angela PISTORIO, Istituto Giannina Gaslini, ITALY
• Clelia ZANABONI, Istituto Giannina Gaslini, ITALY
• Anna GUDDO, Ospedale dei bambini Di Cristina, ITALY (Site Local Investigator)
• Gerald ROGAN NEBA, Ospedale G. Salesi, ITALY (Site Local Investigator)
• Moreno FAVARATO, Ospedale Papa Giovanni XXIII, ITALY
• Bruno Guido LOCATELLI, Ospedale Papa Giovanni XXIII, ITALY (Site Local Investigator)
• Micol MAFFIOLETTI, Ospedale Papa Giovanni XXIII, ITALY
• Valter SONZOGNI, Ospedale Papa Giovanni XXIII, ITALY
• Rossella GARRA, Fondazione Policlinico A.Gemelli IRCCS- Università Cattolica del Sacro Cuore- Roma, ITALY
• Maria SAMMARTINO, Fondazione Policlinico A.Gemelli IRCCS- Università Cattolica del Sacro Cuore- Roma, ITALY (Site Local Investigator)
• Fabio SBARAGLIA, Fondazione Policlinico A.Gemelli IRCCS- Università Cattolica del Sacro Cuore- Roma, ITALY
• Andrea CORTEGIANI, Policlinico P. Giaccone. University of Palermo, ITALY (Site Local Investigator)
• Alessandra MOSCARELLI, Policlinico P. Giaccone. University of Palermo, ITALY
• Elena ATTANASI, Section of Anesthesiology, Analgesia and Intensive Care, ITALY
• Simonetta TESORO, Section of Anesthesiology, Analgesia and Intensive Care, ITALY (Site Local Investigator)
• Cristina AGAPITI, Spedali Civili di Brescia, ITALY (Site Local Investigator)
• Francesca PINZONI, Spedali Civili di Brescia, ITALY
• Cesare VEZZOLI, Spedali Civili di Brescia, ITALY
• Federico BILLOTTA, University of Rome "Sapienza", Policlinico Umberto I, Rome, ITALY (Site Local Investigator)

**LATVIA**
• Arta BARZDINA, Children Clinical University Hospital, LATVIA
• Zane STRAUME, Children Clinical University Hospital, LATVIA (Site Local Investigator, National Coordinator)
• Anda ZUNDANE, Children Clinical University Hospital, LATVIA

**LITHUANIA**
• Laura LUKOSIENĖ, Lithuanian University of Health Sciences Medical Academy, LITHUANIA (Site Local Investigator, National Coordinator)
• Irena MARAULAITE, Lithuanian University of Health Sciences Medical Academy, LITHUANIA
• Ilona RAZLEVICE, Lithuanian University of Health Sciences Medical Academy, LITHUANIA

**LUXEMBOURG**
• Bernd SCHMITZ, Centre Hospitalier de Luxembourg, LUXEMBOURG (Site Local Investigator, National Coordinator)

**MALTA**
• Stephanie MIFSUD, Mater Dei Hospital, MALTA (Site Local Investigator, National Coordinator)

**NETHERLANDS**
• Carolin AEHLING, Amsterdam University Medical Center, University of Amsterdam, NETHERLANDS
• Celia ALLISON, Amsterdam University Medical Center, University of Amsterdam, NETHERLANDS
• Rients DE BOER, Amsterdam University Medical Center, University of Amsterdam, NETHERLANDS
• Dina EMAL, Amsterdam University Medical Center, University of Amsterdam, NETHERLANDS
• Markus STEVENS, Amsterdam University Medical Center, University of Amsterdam, NETHERLANDS (Site Local Investigator)
• Marielle BUITENHUIS, Erasmus MC- Sophia Children's Hospital, NETHERLANDS
• Jurgen DE GRAAFF, Erasmus MC- Sophia Children's Hospital, NETHERLANDS (National Coordinator)
• Inge DE LIEFDE, Erasmus MC- Sophia Children's Hospital, NETHERLANDS
• Andreas MACHOTTA, Erasmus MC- Sophia Children's Hospital, NETHERLANDS
• Gail SCOONES, Erasmus MC- Sophia Children's Hospital, NETHERLANDS
• Lonneke STAALS, Erasmus MC- Sophia Children's Hospital, NETHERLANDS (Site Local Investigator)
• Jeremy TOMAS, Erasmus MC- Sophia Children's Hospital, NETHERLANDS
• Anouk VAN DER KNIJFF-VAN DORTMONT, Erasmus MC- Sophia Children's Hospital, NETHERLANDS
• Marianne VELDHUIZEN, Haga Teaching Hospital, NETHERLANDS (Site Local Investigator)
• David ALDERS, Leiden University Medical Center, NETHERLANDS (Site Local Investigator)
• Wolfgang BUHRE, MUMC, NETHERLANDS
• Eva SCHAFRAT, MUMC, NETHERLANDS
• Jan SCHREIBER, MUMC, NETHERLANDS
• Petronella MARI VERMEULEN, MUMC, NETHERLANDS (Site Local Investigator)
• Mark HENDRIKS, Radboudumc, NETHERLANDS (Site Local Investigator)
• Sandra LAKO, Radboudumc, NETHERLANDS
• Marieke VOET-LINDNER, Radboudumc, NETHERLANDS
• Barbe PIETERS, Radboudumc, NETHERLANDS
• Gert-Jan SCHEFFER, Radboudumc, NETHERLANDS
• Luc TIELENS, Radboudumc, NETHERLANDS
• Anthony R. ABSALOM, Universitair Medisch Centrum Groningen, NETHERLANDS
• Margot BERGSMA, Universitair Medisch Centrum Groningen, NETHERLANDS
• Joke DE RUITER, Universitair Medisch Centrum Groningen, NETHERLANDS
• Sascha MEIER, Universitair Medisch Centrum Groningen, NETHERLANDS
• Martin VOLKERS, Universitair Medisch Centrum Groningen, NETHERLANDS (Site Local Investigator)
• Tjerk ZWEERS, Universitair Medisch Centrum Groningen, NETHERLANDS
• Anne M. BEUKERS, Amsterdam University Medical Center, VU University, NETHERLANDS
• Christa BOER, Amsterdam University Medical Center, VU University, NETHERLANDS (Site Local Investigator)
• Jurgen DERTINGER, Amsterdam University Medical Center, VU University, NETHERLANDS
• Sandra NUMAN, University Medical Center Utrecht, NETHERLANDS
• Bas VAN ZAANE, University Medical Center Utrecht, NETHERLANDS (Site Local Investigator)

**NORWAY**
• Wenche B BOERKE, Oslo University Hospital, Rikshospitalet, NORWAY (Site Local Investigator, National Coordinator)
• Nil EKIZ, Oslo University Hospital, Rikshospitalet, NORWAY
• Kristoffer STENSRUD, Oslo University Hospital, Rikshospitalet, NORWAY
• Inger Marie DRAGE, Oslo University Hospital-Ullevål, NORWAY (Site Local Investigator)
• Erik Ramon ISERN, St. Olavs Hospital, Trondheim University Hospital, NORWAY (Site Local Investigator)

**POLAND**
• Alicja BARTKOWSKA-SNIATKOWSKA, Poznan University of Medical Sciences, Department of Pediatric Anesthesiology and Intensive Care, POLAND (Site Local Investigator)
• Malgorzata GRZESKOWIAK, Poznan University of Medical Sciences, Department of Pediatric Anesthesiology and Intensive Care, POLAND
• Magdalena JUZWA-SOBIERAJ, Poznan University of Medical Sciences, Department of Pediatric Anesthesiology and Intensive Care, POLAND
• Jowita ROSADA-KURASIŃSKA, Poznan University of Medical Sciences, Department of Pediatric Anesthesiology and Intensive Care, POLAND
• Artur BARANOWSKI, Public Paediatric Teaching Hospital of Medical University of Warsaw, POLAND
• Karina JAKUBOWSKA, Public Paediatric Teaching Hospital of Medical University of Warsaw, POLAND
• Dorota LEWANDOWSKA, Public Paediatric Teaching Hospital of Medical University of Warsaw, POLAND
• Magdalena MIERZEWSKA-SCHMIDT, Public Paediatric Teaching Hospital of Medical University of Warsaw, POLAND (Site Local Investigator)
• Piotr SAWICKI, Public Paediatric Teaching Hospital of Medical University of Warsaw, POLAND
• Magdalena URBAN-LECHOWICZ, Public Paediatric Teaching Hospital of Medical University of Warsaw, POLAND
• Pomianek PRZEMYSLAW, Wroclaw Medical University, POLAND
• Marzena ZIELINSKA, Wroclaw Medical University, POLAND (Site Local Investigator, National Coordinator)

**PORTUGAL**
• Teresa LEAL, Centro Hospitalar do Porto, PORTUGAL
• Maria SOARES, Centro Hospitalar do Porto, PORTUGAL
• Pedro PINA, Centro Hospitalar do Porto, PORTUGAL (Site Local Investigator)
• Sílvia PINHO, Centro Hospitalar do Porto, PORTUGAL
• Maria Domingas PATULEIA, Centro Hospitalar Lisboa Norte - Hospital de Santa Maria, PORTUGAL (Site Local Investigator, National Coordinator)
• Catarina CRUZ ESTEVES, Centro Hospitalar de Lisboa Central - Hospital D. Estefânia, PORTUGAL (Site Local Investigator)
• Helena SALGADO, Hospital de Braga, PORTUGAL (Site Local Investigator)
• Maria João SANTOS, Hospital de Braga, PORTUGAL

**ROMANIA**
• Rodica BADETI, CHILDREN HOSPITAL 'LOUIS TURCANU", ROMANIA (Site Local Investigator)
• Iulia CINDEA, Emergency Clinical Hospital of Constanta, ROMANIA (Site Local Investigator)
• Loredana OANA, Emergency Hospital for Children Cluj Napoca, ROMANIA (Site Local Investigator)
• Adriana GURITA, Spitalul de copii "Maria Curie", ROMANIA
• Luminita ILIE, Spitalul de copii "Maria Curie", ROMANIA
• Gabriel MOCIOIU, Spitalul de copii "Maria Curie", ROMANIA
• Irina TRANTE, Spitalul de copii "Maria Curie", ROMANIA
• Radu TABACARU, Spitalul de copii "Maria Curie", ROMANIA (Site Local Investigator)
• Valentin MUNTEANU, St. Maria" Children's Hospital, ROMANIA (Site Local Investigator)
• Mihai MORARIU, Tirgu Mures Clinical Emergency Hospital, ROMANIA (Site Local Investigator)
• Emese NYÍRI, Tirgu Mures Clinical Emergency Hospital, ROMANIA

**SERBIA**
• Ivana BUDIC, Clinic for Anesthesiology and Intensive Therapy, Clinical Centre Nis, Medical Faculty University of Nis, SERBIA (Site Local Investigator)
• Vesna MARJANOVIC, Clinic for Anesthesiology and Intensive Therapy, Clinical Centre Nis, Medical Faculty University of Nis, SERBIA
• Biljana DRASKOVIĆ, Children and Youth Health Care Institute of Vojvodina, SERBIA
• Marina PANDUROV, Children and Youth Health Care Institute of Vojvodina, SERBIA
• Jordanka ILIC, Mother and Child Healthcare Institute of Serbia, SERBIA
• Ana MANDRAS, Mother and Child Healthcare Institute of Serbia, SERBIA
• Zdenka RADOS, Mother and Child Healthcare Institute of Serbia, SERBIA
• Nikola STANKOVIC, Mother and Child Healthcare Institute of Serbia, SERBIA (Site Local Investigator)
• Maja SUICA, Mother and Child Healthcare Institute of Serbia, SERBIA
• Sladjana VASILJEVIC, Mother and Child Healthcare Institute of Serbia, SERBIA
• Mirjana KNEZEVIC, University Children s Hospital, Belgrade, SERBIA
• Irina MILOJEVIC, University Children s Hospital, Belgrade, SERBIA (Site Local Investigator)
• Ivana PETROV, University Children s Hospital, Belgrade, SERBIA
• Selena PURIC RACIC, University Children s Hospital, Belgrade, SERBIA
• Dusica SIMIC, University Children s Hospital, Belgrade, SERBIA (National Coordinator)
• Irena SIMIC, University Children s Hospital, Belgrade, SERBIA
• Marija STEVIC, University Children s Hospital, Belgrade, SERBIA
• Irena VULICEVIC, University Children s Hospital, Belgrade, SERBIA

**SLOVAKIA**
• Barbora CABANOVÁ, University Children hospital, Banská Bystrica, SLOVAKIA (Site Local Investigator)
• Miloslav HANULA, University Children hospital, Banská Bystrica, SLOVAKIA (National Coordinator)

**SLOVENIA**
• Jelena BERGER, University Clinical Centre Ljubljana, SLOVENIA (Site Local Investigator, National Coordinator)
• Darja JANJATOVIC, University Clinical Centre Ljubljana, SLOVENIA
• Špela PIRTOVŠEK ŠTUPNIK, University Clinical Centre Ljubljana, SLOVENIA

**SPAIN**
• Dolores MÉNDEZ, Hospital 12 de Octubre, SPAIN (Site Local Investigator)
• Gema PINO, Hospital 12 de Octubre, SPAIN
• Paloma RUBIO, Hospital 12 de Octubre, SPAIN
• Silvia LÓPEZ, Hospital Universitari Parc Taulí Sabadell, SPAIN (Site Local Investigator)
• Alberto IZQUIERDO, Hospital Universitari Parc Taulí Sabadell, SPAIN
• Cristina GONZÁLEZ SERRANO, Hospital Universitario Donostia, SPAIN (Site Local Investigator)
• Jesús CEBRIÁN, Hospital General Universitario Gregorio Marañón, SPAIN (Site Local Investigator)
• Ana PELETEIRO, Hospital General Universitario Gregorio Marañón, SPAIN
• Ernesto MARTÍNEZ GARCÍA, Hospital Infantil Universitario Niño Jesús, SPAIN (Site Local Investigator)
• Pilar DEL REY DE DIEGO, Hospital Infantil Universitario Niño Jesús, SPAIN
• Carolina TORMO DE LAS HERAS, Hospital Infantil Universitario Niño Jesús, SPAIN
• Pablo TRONCOSO MONTERO, Hospital Infantil Universitario Niño Jesús, SPAIN
• Montserrat SUAREZ COMAS, Hospital Sant Joan De Déu, SPAIN (Site Local Investigator)
• Celia ARBONA, Hospital Sant Joan De Déu, SPAIN
• David ARTÉS, Hospital Sant Joan De Déu, SPAIN
• Alicia CHAMIZO, Hospital Sant Joan De Déu, SPAIN
• Silvia SERRANO, Hospital Sant Joan De Déu, SPAIN
• Francisco ESCRIBÁ, Hospital Universitari i Politècnic la Fe, SPAIN (Site Local Investigator)
• Cristina AULI, Hospital Universitari Son Espases, SPAIN (Site Local Investigator)
• Ceferina SUÁREZ CASTAÑO, Hospital Universitario Marqués de Valdecilla, SPAIN (Site Local Investigator)
• Osvaldo PÉREZ PARDO, Hospital Universitario Marqués de Valdecilla, SPAIN
• Natalia SIERRA BIDDLE, Hospital Universitario Marqués de Valdecilla, SPAIN
• María ISABEL VILLALOBOS RICO, Hospital Universitario Marqués de Valdecilla, SPAIN
• Irene GARCÍA MARTÍNEZ, Vall d'Hebron Barcelona Hospital Campus, SPAIN (Site Local Investigator)
• Susana MANRIQUE MUÑOZ, Vall d'Hebron Barcelona Hospital Campus, SPAIN
• Nuria MONTFERRER ESTRUCH, Vall d'Hebron Barcelona Hospital Campus, SPAIN
• Elena VILARDELL ORTÍZ, Vall d'Hebron Barcelona Hospital Campus, SPAIN
• Rodrigo POVES-ÁLVAREZ, Hospital Clínico Universitario de Valladolid, SPAIN

**SWEDEN**
• Ivan KOHN, Astrid Lindgren Children's Hospital, SWEDEN (Site Local Investigator)
• Ulf LINDESTAM, Astrid Lindgren Children's Hospital, SWEDEN
• Jarl REINHARD, Astrid Lindgren Children's Hospital, SWEDEN
• Albert CASTELLHEIM, Queen Silvia Children´s Hospital, Sahlgrenska University Hospital, SWEDEN (Site Local Investigator, National Coordinator)
• Kerstin SANDSTRÖM, Queen Silvia Children´s Hospital, Sahlgrenska University Hospital, SWEDEN
• Peter FRYKHOLM, Uppsala University Hospital, SWEDEN (Site Local Investigator)
• Sporre BENGT, Uppsala University Hospital, SWEDEN
• Rainer DÖRENBERG, Uppsala University Hospital, SWEDEN
• Maria GARCIA, Uppsala University Hospital, SWEDEN
• Ann KVARNSTRÖM, Uppsala University Hospital, SWEDEN
• Emma PONTÉN, Uppsala University Hospital, SWEDEN

**SWITZERLAND**
• Thomas BRUELISAUER, Bern University Hospital, SWITZERLAND
• Gabor ERDOES, Bern University Hospital, SWITZERLAND
• Heiko KAISER, Bern University Hospital, SWITZERLAND
• Mathias MARCHON, Bern University Hospital, SWITZERLAND
• Thomas RIVA, Bern University Hospital, SWITZERLAND
• Stefan SEILER, Bern University Hospital, SWITZERLAND (Site Local Investigator)
• Yann BÖGLI, Centre Hospitalier Universitaire Vaudois, SWITZERLAND
• Mirko DOLCI, Centre Hospitalier Universitaire Vaudois, SWITZERLAND (Site Local Investigator)
• Carine MARCUCCI, Centre Hospitalier Universitaire Vaudois, SWITZERLAND
• Walid HABRE, Geneva Children's Hospital, SWITZERLAND (Site Local Investigator, National Coordinator)
• Isabelle PICHON, Geneva Children's Hospital, SWITZERLAND
• Laszlo VUTSKITS, Geneva Children's Hospital, SWITZERLAND
• Mattias CASUTT, Kantonsspital Luzern, SWITZERLAND
• Martin HÖLZLE, Kantonsspital Luzern, SWITZERLAND
• Thomas HURNI, Kantonsspital Luzern, SWITZERLAND
• Martin JÖHR, Kantonsspital Luzern, SWITZERLAND
• Anna-Ursina MALÄR, Kantonsspital Luzern, SWITZERLAND
• Jacqueline MAUCH, Kantonsspital Luzern, SWITZERLAND (Site Local Investigator)
• Thomas ERB, University Children's Hospital Basel, SWITZERLAND (Site Local Investigator)
• Karin OEINCK, University Children's Hospital Basel, SWITZERLAND

**TURKEY**
• Mine AKIN, Ankara Children's Health and disease Haematology Oncology Training And Research Hospital, TURKEY
• Gulsen KESKIN, Ankara Children's Health and disease Haematology Oncology Training And Research Hospital, TURKEY
• Yesim SENAYLI, Ankara Children's Health and disease Haematology Oncology Training And Research Hospital, TURKEY (Site Local Investigator)
• Guner KAYA, Cerrahpasa Medical School, TURKEY
• Pinar KENDIGELEN, Cerrahpasa Medical School, TURKEY
• Ayse Çiğdem TUTUNCU, Cerrahpasa Medical School, TURKEY (Site Local Investigator)
• Zehra HATIPOĞLU, Çukurova University, TURKEY
• Dilek ÖZCENGIZ, Çukurova University, TURKEY (Site Local Investigator)
• Hale Aksu ERDOST, Dokuz Eylül University, TURKEY
• Elvan ÖÇMEN, Dokuz Eylül University, TURKEY
• Çimen OLGUNER, Dokuz Eylül University, TURKEY (Site Local Investigator)
• Hilmi AYANOGLU, Marmara University, TURKEY
• Pelin Corman DINCER, Marmara University, TURKEY
• Tumay UMUROGLU, Marmara University, TURKEY (Site Local Investigator)
• Mustafa AZİZOĞLU, Mersin University - Anaesthesiology, TURKEY
• Handan BIRBİÇER, Mersin University - Anaesthesiology, TURKEY (Site Local Investigator)
• Nurcan DORUK, Mersin University - Anaesthesiology, TURKEY
• Aslı SAGUN, Mersin University - Anaesthesiology, TURKEY
• Sibel BARIS, Ondokuz Mayis University, TURKEY (Site Local Investigator)

**UKRAINE**
• Dmytro DMYTRIIEV, Vinnytsia National Medical University, UKRAINE (Site Local Investigator, National Coordinator)

**UNITED KINGDOM**
• Sridevi KUCHI, Alder Hey Children's Hospital, UNITED KINGDOM (Site Local Investigator)
• Nuria MASIP, Alder Hey Children's Hospital, UNITED KINGDOM
• Peter BROOKS, Chelsea & Westminster Hospital, UNITED KINGDOM (Site Local Investigator)
• Alison HARE, Chelsea & Westminster Hospital, UNITED KINGDOM
• Nargis AHMAD, Great Ormond St Hospital, UNITED KINGDOM
• Michelle CASEY, Great Ormond St Hospital, UNITED KINGDOM
• Sam DE SILVA, Great Ormond St Hospital, UNITED KINGDOM
• Nadine DOBBY, Great Ormond St Hospital, UNITED KINGDOM
• Prakash KRISHNAN, Great Ormond St Hospital, UNITED KINGDOM
• L. Amaki SOGBODJOR, Great Ormond St Hospital, UNITED KINGDOM
• Ellie WALKER, Great Ormond St Hospital, UNITED KINGDOM
• Suellen WALKER, Great Ormond St Hospital, UNITED KINGDOM (Site Local Investigator, National Co‐Coordinator)
• Stephanie KING, Guy's and St Thomas's NHS Trust, UNITED KINGDOM
• Katy NICHOLSON, Guy's and St Thomas's NHS Trust, UNITED KINGDOM (Site Local Investigator)
• Michelle QUINNEY, Guy's and St Thomas's NHS Trust, UNITED KINGDOM
• Paul STEVENS, Guy's and St Thomas's NHS Trust, UNITED KINGDOM
• Andrew BLEVIN, King's College Hospital, UNITED KINGDOM
• Mariangela GIOMBINI, King's College Hospital, UNITED KINGDOM
• Chulananda GOONASEKERA, King's College Hospital, UNITED KINGDOM (Site Local Investigator)
• Sadia ADIL, Leeds Children's Hospital, UNITED KINGDOM
• Stephanie BEW, Leeds Children's Hospital, UNITED KINGDOM
• Carol BODLANI, Leeds Children's Hospital, UNITED KINGDOM
• Dan GILPIN, Leeds Children's Hospital, UNITED KINGDOM
• Stephanie JINKS, Leeds Children's Hospital, UNITED KINGDOM
• Nalini MALARKKAN, Leeds Children's Hospital, UNITED KINGDOM
• Alice MISKOVIC, Leeds Children's Hospital, UNITED KINGDOM
• Rebecca PAD, Leeds Children's Hospital, UNITED KINGDOM
• Juliet Wolfe BARRY, Leeds Children's Hospital, UNITED KINGDOM (Site Local Investigator)
• Joy ABBOTT, Nottingham University Hospital, UNITED KINGDOM
• James ARMSTRONG, Nottingham University Hospital, UNITED KINGDOM (Site Local Investigator)
• Natalie COOPER, Nottingham University Hospital, UNITED KINGDOM
• Lindsay CRATE, Nottingham University Hospital, UNITED KINGDOM
• John EMERY, Nottingham University Hospital, UNITED KINGDOM
• Kathryn JAMES, Nottingham University Hospital, UNITED KINGDOM
• Hannah KING, Nottingham University Hospital, UNITED KINGDOM
• Paul MARTIN, Nottingham University Hospital, UNITED KINGDOM
• Stefano SCALIA CATENACCI, Royal Aberdeen Children's Hospital, UNITED KINGDOM (Site Local Investigator)
• Rob BOMONT, Royal Alexandra Childrens' Hospital, UNITED KINGDOM
• Paul SMITH, Royal Alexandra Childrens' Hospital, UNITED KINGDOM (Site Local Investigator)
• Sara MELE, Royal Brompton Hospital, UNITED KINGDOM (Site Local Investigator)
• Alessandra VERZELLONI, Royal Brompton Hospital, UNITED KINGDOM
• Philippa DIX, Royal Devon and Exeter NHS Foundation Trust, UNITED KINGDOM (Site Local Investigator)
• Graham BELL, Royal Hospital for Sick Children Glasgow, UNITED KINGDOM (Site Local Investigator)
• Elena GORDEVA, Royal Hospital for Sick Children Glasgow, UNITED KINGDOM
• Lesley MCKEE, Royal Hospital for Sick Children Glasgow, UNITED KINGDOM
• Esther NGAN, Royal Hospital for Sick Children Glasgow, UNITED KINGDOM
• Jutta SCHEFFCZIK, Royal Hospital for Sick Children Glasgow, UNITED KINGDOM
• Li-En TAN, Royal Hospital for Sick Children Glasgow, UNITED KINGDOM
• Mark WORRALL, Royal Hospital for Sick Children Glasgow, UNITED KINGDOM
• Carmel CASSAR, Royal London Hospital, UNITED KINGDOM (Site Local Investigator)
• Kevin GODDARD, Royal London Hospital, UNITED KINGDOM
• Victoria BARLOW, Royal Manchester Children's Hospital, UNITED KINGDOM
• Vimmi OSHAN, Royal Manchester Children's Hospital, UNITED KINGDOM (Site Local Investigator)
• Khairi SHAH, Royal Manchester Children's Hospital, UNITED KINGDOM
• Sarah BELL, Royal Victoria Infirmary, UNITED KINGDOM
• Lisa DANIELS, Royal Victoria Infirmary, UNITED KINGDOM
• Monica GANDHI, Royal Victoria Infirmary, UNITED KINGDOM (Site Local Investigator)
• David PACHTER, Royal Victoria Infirmary, UNITED KINGDOM
• Chris PERRY, Royal Victoria Infirmary, UNITED KINGDOM
• Andrew ROBERTSON, Royal Victoria Infirmary, UNITED KINGDOM
• Carmen SCOTT, Royal Victoria Infirmary, UNITED KINGDOM
• Lynne WARING, Royal Victoria Infirmary, UNITED KINGDOM
• David BARNES, St George’s University Hospitals NHS Foundation Trust, UNITED KINGDOM
• Sophie CHILDS, St George’s University Hospitals NHS Foundation Trust, UNITED KINGDOM
• Joanne NORMAN, St George’s University Hospitals NHS Foundation Trust, UNITED KINGDOM
• Robin SUNDERLAND, St George’s University Hospitals NHS Foundation Trust, UNITED KINGDOM (Site Local Investigator)

**Management Team**
• European Society of Anaesthesiology and Intensive Care Medicine, Brussels: Dowell JULIA; Feijten PRISCA; Harlet PIERRE; Herbineaux SARAH; Leva BRIGITTE; Plichon BENOÎT; Virág KATALIN
